# Line-dropped gelatin multi-element calibration standards in LA-ICP-MS: a statistically verifying comparison with cryosectioned homogenized lung and liver as matrix-matched calibration standards and as corresponding reference materials

**DOI:** 10.1007/s44211-024-00691-8

**Published:** 2024-11-27

**Authors:** Sven Thoröe-Boveleth, Ruth Becker, Jens Bertram, Thomas Schettgen, Manfred Möller, Danny Jonigk, Thomas Kraus, Ralf Weiskirchen

**Affiliations:** 1 Institute for Occupational, Social and Environmental Medicine (IASU), Medical Faculty, RWTH University Hospital Aachen, Pauwelsstraße 30, 52074 Aachen, Germany; 2 Institute of Molecular Pathobiochemistry, Experimental Gene Therapy and Clinical Chemistry (IFMPEGKC), Medical Faculty, RWTH University Hospital Aachen, Pauwelsstraße 30, 52074 Aachen, Germany; 3 Institute of Pathology, Medical Faculty, RWTH University Hospital Aachen, Pauwelsstraße 30, 52074 Aachen, Germany; 4 German Center for Lung Research (DZL), Biomedical Research in End-Stage and Obstructive Lung Disease Hannover (BREATH), Hanover, Germany

**Keywords:** VBA, Gelatin, Tissue homogenates, Calibration, LA-ICP-MS

## Abstract

Calibrations in LA-ICP-MS are typically very time-consuming and complex, as they need to be matched to the samples being measured and sectioned on a microtome. Alternatively, gelatin can be in droplet form or as a section, which is a more recent development. In this study, we report on investigations where hot multi-element gelatin solutions are placed in a linear fashion on microscopic slides to conduct comparative statistical observations between doped tissue homogenates from the liver and lung. The tissue homogenates served as both samples (complete ablation) and calibration standards (partial ablation) for verification purposes. We explored the effects of different laser fluences (0.50–1.50 J/cm^2^), gelatin contents (0.3–20.0%) and section thicknesses (10–30 µm). To do this, we evaluated the samples by calculating median and mean values over the entire section with and without removal of elementary spikes (de-spiking). A reduction in distribution was achieved with averaging. The data was normalized using ^13^C as an internal standard. In these experiments and under these measurement conditions, it was observed that the selected laser fluences, gelatin contents, and section thicknesses did not visibly affect the results, making them comparable. Each sample could be assessed with each gelatin calibration, allowing for determination of expected reference values. Despite interruptions in the measurement operation, due to the high number of measurements, where samples and calibrations could not be analyzed in one measurement run, no negative effects of stopping and starting the LA-ICP-MS were observed.

**Graphical abstract:**

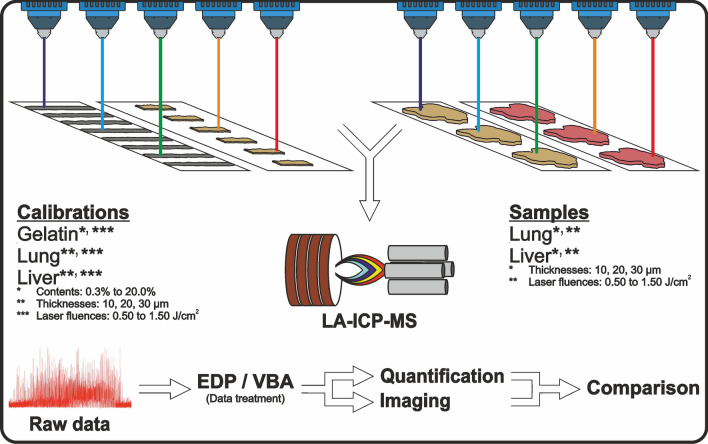

**Supplementary Information:**

The online version contains supplementary material available at 10.1007/s44211-024-00691-8.

## Introduction

LA-ICP-MS (laser ablation inductively coupled plasma mass spectrometry) has been a technique with constantly growing fields of application since the early 1990s [1–4]. Today it is considered one of the most versatile yet challenging method in analytics. The evaluation process is complex, often requiring the use of proprietary software solutions. This methods finds applications in various fields such as life sciences (e.g. biology [5, 6], medicine [7–11], biomedicine [12–14]), geology [15–17], forensics [18–20] and archeology [21–23], among others. In the field of life sciences, there are numerous quantification methods available. In addition, there are various methods for utilizing elements as internal standards, even if they are not naturally present in the tissue. As a result, there are multiple techniques for introducing these elements into the tissue for analysis. This can be achieved, for example, by applying a film to a slide containing the corresponding internal standard [24–26]. The tissue section to be examined is then placed on this film. Another option is to sputter the internal standard onto a tissue section already on the slide as a fine dust [27, 28]. In both cases, the internal standard should be replicated in the correct ratio to the analytes during the measurement. However, most working groups use the ^13^C isotope as the internal standard, as it is already present in the biologic materials and is considered to be relatively homogeneously distributed [29–35]. The ^34^S isotope is also used as an internal standard [36–38]. Both provide a sufficiently strong and sensitive signal, which eliminates many work steps and reduces the workload.

Many studies also describe the use of the tissues to be analyzed for standard production (matrix-matched standards) [39–43]. This process involves a significant amount of work, as the carrier material must be finally homogenized and the dry mass determined in order to add the individual elements proportionally [44–47]. These elements are then added as a solution to the non-dry material. To prepare the doped homogenized material for sectioning later, it is advisable to remove a large portion of the present water (doping solution, tissue) before freezing at − 80 °C. The use of this method is on the decline, however. In more recent work, gelatin is used for the production of these standards instead of the materials to be analyzed [48–52]. Gelatin contains ^13^C and ^34^S in comparable quantities, but is easier to produce as it can be doped in liquid form. However, both methods still require a cryomicrotome for histology.

A further optimization of this newer method does not require a microtome. Instead, the doped liquid gelatin solution, first described by Šala et al. [53], is dropped onto a microscope slide. This can be done using pipettes, specially prepared inkjet printers, or in special molds [54–58].

The purpose of this study is to develop a simple, fast and universal calibration method that can be used multiple times. This will be achieved by arranging individual calibration standards in a linear arrangement, eliminating the need for new calibration for each ablation procedure. The method used in this study is based on Šala et al. [53] but aims to provide more comprehensive statistical evaluations. This includes examining the effects of different laser fluences, sample section thicknesses, and gelatin content of the gelatin calibrations. The observations from this study will then be compared with results obtained using established methods of conventional matrix-matched calibrations. The samples will be analyzed with each individual calibration and presented in box-whisker plots. Results will be shown as both median and mean values over the entire section, with and without the removal of elemental spikes.

## Materials and methods

### Chemicals and materials

Aluminum nitrate nonahydrate, ortho-phosphoric acid, as well as As, Be, Cd, Co, Mn, Pt, V, Y and Zr were purchased as 1 g/L ICP-MS standards and Hg as a 10 g/L solution from Merck, Darmstadt, Germany. Furthermore, we also purchased Rh as 10 mg/L solution (IS), nitric acid (suprapure, 65%), hydrochloric acid (suprapure, 32%) and hydrogen peroxide (suprapure, 30%) also from Merck, Darmstadt, Germany. Calcium nitrate tetrahydrate and ammonium dimolybdate were obtained from Roth, Karlsruhe, Germany. The salts chromium(III) nitrate nonahydrate, copper(II) nitrate trihydrate, iron(III) nitrate nonahydrate, potassium nitrate, magnesium nitrate hexahydrate, sodium nitrate, nickel(II) nitrate hexahydrate, and zinc nitrate hexahydrate were purchased from Sigma-Aldrich, St. Louis, USA, and also the gelatin from pig skin, type A, with a bloom strength of 300. The chicken liver was purchased from an organic butcher, and the bovine lung (from a young bull) comes from a farm that is not certified organic but adheres these standards. The standard reference materials Whole Milk Powder (NIST-SRM-1549a) and Bovine liver (NIST-SRM-1577C) from the National Institute of Standards and Technology (NIST) were used as verification materials. Both materials were received from LGC, Teddington, England. The Eppendorf pipette tips (epT.I.P.S) were purchased in sizes 0.1–10 µL, 2–200 µL and 50–1000 µL from Eppendorf, Hamburg, Germany.

#### Preparation of the calibration materials from tissue homogenates and the samples

Individual salts were dissolved near their maximum solubility to create a highly concentrated mixed solution, minimizing the volume of liquid added to the homogenized samples. To compare two different types of tissue, liver and lung samples were selected. The samples of both tissues were then homogenized in the OmniBlendV high-performance mixer (Naassan Com GmbH, Essen, Germany) and the dry mass was determined using the HB43 halogen moisture meter (Mettler-Toledo, Giessen, Germany). This step is essential for calculating the volumes of highly concentrated mixing solution to be added based on the dry mass. Final concentrations for As, Be, Cd, Co, Cr, Cu, Hg, Mn, Mo, Ni, Pt, V, Y and Zr of 0 µg/g (homogenized material without spiking, S0) and 1, 2, 5, 8, 10 µg/g (S1–S5) were targeted, as these elements are not expected to occur in tissues, or only in very low concentrations. A tenfold higher concentration was chosen for Al and Zn (expectably higher concentrated in tissues), a 100-fold higher concentration for Ca, Fe and Mg and a thousand times the initial concentration for K, P, and Na (highest expected concentrations). The chemical elements used were selected on the bases of their natural occurrence in biologic samples, but also on the other hand on the potency of their toxic effects and the common use in our laboratory. This protocol was applied to both tissue materials. All homogenized samples (S0–S5) were then carefully but thoroughly homogenized and spread on microscope slides (Knittel Glass, Braunschweig, Germany) to remove excess water (over several hours). To prevent uneven drying, the mass was gently shifted several times per hour using a glass rod. Once the mixture became moldable at room temperature, it was frozen in cuboid blocks at − 80 °C for easier cutting with a cryo-microtome at a later stage. Three pieces (100–200 mg) were isolated from each homogenized material (S0–S5) and reserved for the ICP-MS analysis. Sections of the remaining material were prepared using a CM1950 cryomicrotome (Leica Biosystems, Wetzlar, Germany). The cryomicrotome operated at a cryochamber temperature of − 21 °C and an object area temperature of − 18 °C. Three calibrations were generated for each material (lung, liver) following the pattern S0, S1, …, S5 at 10, 20 and 30 µm each and placed on separate slides. In addition, 30 sections per material (lung, liver) were prepared from the block with the standard S4 (8; 80; 800; 8000 µg/g) in thicknesses of 10, 20 and 30 µm. Ten sections were prepared of each thickness and placed in the highest possible quantity of sections per slide. Each material was to be ablated as a double determination (*n* = 2) with each thickness at five different laser fluences (0.5, 0.75, 1.00, 1.25 and 1.50 J/cm^2^). The calibration and sample sections were air-dried and stored at room temperature until analyzed. Various thicknesses of tissue sections and different laser fluences were selected to investigate the potential effects in this comparative study.

#### Preparation of the gelatin calibration materials

The protocol closely follows that of Suárez-Oubiña et al. [58], but it was developed independently. First the dry mass of the gelatin powder was determined. Subsequently, solutions of 0.3 wt%, 0.5 wt%, 0.7 wt%, 2.0 wt%, 5.0 wt%, 7.0 wt%, 8.0 wt%, 9.0 wt%, 15.0 wt%, 20.0 wt% were prepared in a hot water bath. The standard series were prepared following the above section (2.2.1) with 8 calibration points (S0–S7) including additional concentration ranges of 20–20,000 (S6) and 30–30,000 (S7). In this case, 1 mL gelatin solutions were prepared (final volume), and then doped with a highly concentrated element mixture in a hot state, with volumes defined by their proportion of dry mass. The gelatin-element mix solutions were then cooled as much as possible toward room temperature, but remained pipettable. The next step involved carefully drawing the lines across the microscope slide to minimize chromatography effects (such as coffee-ring effects or Marangoni effects) using a 10-µL Eppendorf pipette with 0.1–10-µL tips. Due to the lack of a suitable convection oven, as recommended by Sala et al. [53], drying at room temperature was chosen. Tests with an oven (without forced convection) resulted in strong zone formation, which was less pronounced with air drying.

Neither the method of Sala et al. [53], the method like that of Braeuer et al. [56] nor the method used in this work can produce exact thicknesses, as the droplet is typically thinner at the edges than in the center. Therefore, careful homogenization and thorough ablation of the material are crucial.

### ICP-MS measurements

To determine the actual contents, the three previously separated homogenized pieces (S0–S5) were each accurately weighed into microwave pressure digestion vessels. The same procedure was used with the standard reference materials Whole Milk Powder (NIST-SRM-1549a) and Bovine liver (NIST-SRM-1577C). The target weight was 200 mg for the measurements (*n* = 3). Subsequently, 1 mL of internal standard (IS, 1 mg Rhodium/L), 2.5 mL of nitric acid, 2.5 mL of hydrochloric acid, 2.5 mL of hydrogen peroxide, and 2.5 mL of MilliQ water were added to each vessel. The samples prepared in this way were then digested in the Ethos.lab microwave pressure digestion system (MLS GmbH, Leutkirch, Germany). In the program used, the samples were first brought to 210 °C within 45 min. This temperature was maintained for 15 min and then cooled to room temperature. After a 1:10 dilution, the samples were analyzed with an ICP-MS (8900-QQQ, Agilent, Waldbronn, Germany). Settings used for ICP-MS are shown in Table 1.

**Table 1:** ICP-MS measurement settings for microwave pressure digestion

ICP-MS settings	
Nebulizer	MicroMist
Nebulizer gas flow	1.15 L/min
Injector	2 mm quarz glass
RF power	1,550 watts
Plasma gas flow	15 L/min
Replicates	3
Sweeps/replicate	20
Scanning mode	Peak hopping
Dwell time	0.5–3.0 s
Analytical procedure*	9:32 min/sample
	5:12 h/run

### LA-ICP-MS measurements

The previously mentioned ICP-MS was used for the LA-ICP-MS measurements with a New Wave NWR213 laser (Elemental Scientific, Omaha, NE, USA) connected to the torch of the ICP-MS. Table 2 shows the used LA-ICP-MS settings.

**Table 2:** ICP-MS and LA settings for LA-ICP-MS

ICP-MS settings	
Carrier gas	Argon
Carrier gas flow	0.95 L/min
Injector	2 mm quarz glass
RF power	1,300 watts
plasma gas flow	15 L/min
Dwell time	≈ 5 ms/element
Cycle time	0.2143 s
Scanning mode	Peak hopping
LA settings	
Ablation gas	Helium
Ablation gas flow	0.8 L/min
Laser spot size	60 µm
Laser profile	radial
Scanning speed	70 µm/s
Ablation mode	Line scan
Repetition frequency	20 Hz
Sensitivity (cps)	≈ 6000^a^ (*n* = 7)
oxide production rate	≈ 0.38%^a^ (*n* = 7)

^a232^Th, NIST SRM 610

**Fig. 1:**
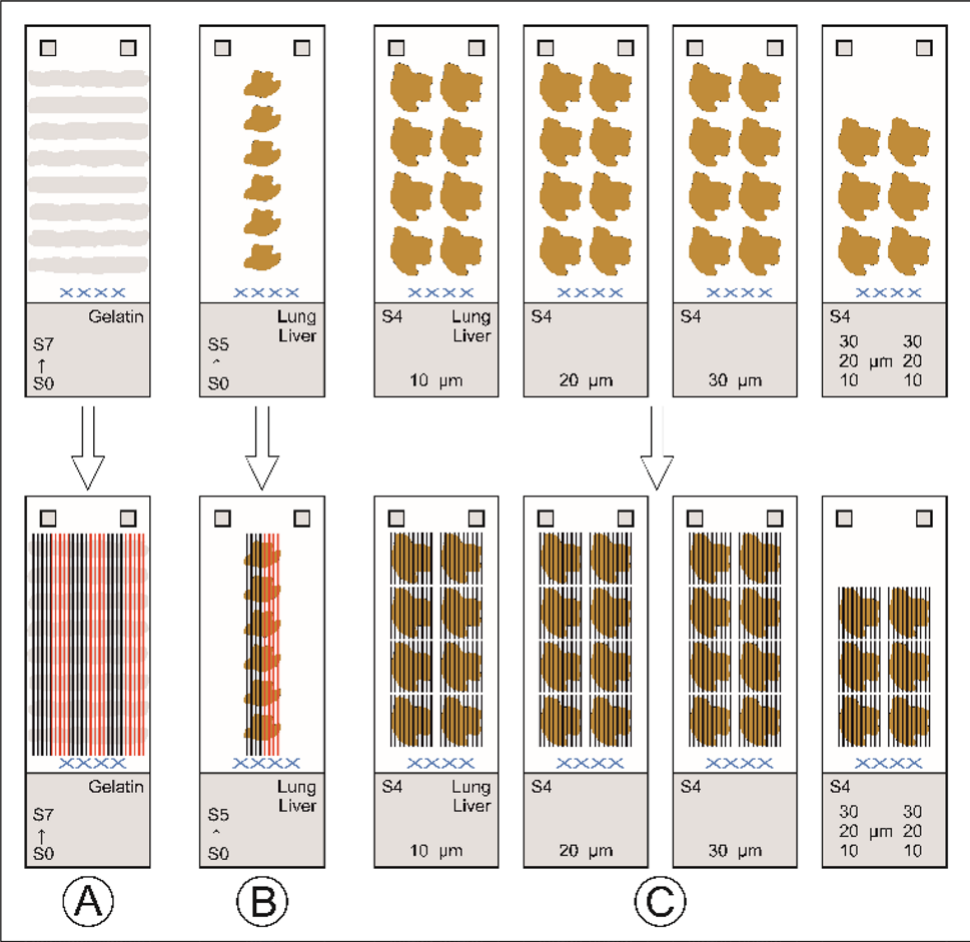
**A** Six calibration sets for gelatin per material concentration (2.2.2). One set includes one calibration for each laser fluence with five ablation lines per calibration, together five calibrations with 25 ablation lines (shown alternately in black and red for better visualization). This results in 30 calibrations per gelatin concentration (2.2.2) with 150 ablation lines. And a total of 300 calibrations with 1500 ablation lines for all measured gelatin concentrations. **B** The same scheme is used for the tissue homogenates (lung, liver). Two calibration sets per section thickness (2.2.1). This results in 10 calibrations per section thickness with 50 ablation lines and a total of 60 (2 × 30) calibrations with 300 ablation lines for all measured section thicknesses. **C** 30 lung tissues (S4) and 30 liver tissues (S4) as samples

For the LA laser fluence of 0.50, 0.75, 1.00, 1.25, 1.50 J/cm^2^ was used. Figure 1 shows the calibration and sample scheme. For gelatin six calibrations were prepared for all gelatin concentrations (2.2.2). For the lung and liver calibrations, two calibration sets were created for each section thickness. This was because the section shapes did not allow six complete sets through all sections of the calibration. In this way, a total of 360 calibrations and 60 samples were measured. Typical ablation/analysis times were approximately 1.5 h per calibration and 4–6 h per sample. In addition, there was a significant computation time and time required for developing the codes.

### Data processing, visualization and statistics

The data evaluation was conducted using VBA scripts based on the Excel Laser Ablation Imaging (ELAI) visualization tool [41, 42, 46, 47], but with specially designed scripts that have not yet been published. However, this is in the planning stage. This includes custom scripts for generating data sets of calibrations and samples (sections), sorting data, and statistically visualizing results using box-whisker plots. In addition, scripts for imaging ablations from calibrations and tissue sections are included.

First, each individual calibration has to be converted from the raw data into an XY table format that can be used for sample calculation. To do this, the section margins (transition from background signals to sample signals and vice versa) of the calibrations (gelatin calibration materials, lung and liver calibration materials) need to be determined, as the ablation lines of the calibrations pass through all sections. The intermediate areas of the calibrations, which only produce background signals, are also evaluated in order to obtain a good background value across the entire ablation lines of each calibration. The section margin detection was realized here using VBA scripts. After all sections have been identified, the next step is to calculate the mean value of each section of the calibration and subtract the mean value of all non-section areas. The intensities of the analytes are then divided by the intensities of the internal standard (IS) to form the analyte–IS ratio (*Y* values). The *X*-values were determined using microwave pressure digestion coupled with ICP-MS and represent the reference values of the respective calibration sections, following the principle of the standard addition method.

The sample calculation (tissue homogenates) is carried out in the same way. In this case too, the section margins are determined in order to determine the mean values of the non-section areas and to characterize their positions so that no calculations take place in these areas, thus saving calculation time. After subtracting the mean of all non-sectional areas from all measurement values, the analyte–IS ratios can be calculated using the XY table of the calibration. This process must be carried out for each individual data point. The median and mean value for the entire section is then determined for each measured element. A maximum value is first defined for the following de-spiking (elemental spikes), which is 2 times the 95th percentile of each ablation line. If a value is higher, it is reduced to this value. In many empirical tests conducted by ourselves, this procedure (code not yet published) has proven to be the most suitable for removing elemental spikes in samples, without affecting the structure of the section. This procedure is then applied to the previously determined median and mean values. As soon as all median and mean values with and without de-spiking have been calculated and saved in the individual result files, all median and mean values are extracted using VBA code. VBA code is then used to sort and group these values and transfer them into large data tables from which overview tables and box-whisker plots can be generated, also using VBA code. A data table is created for each element/isotope and sample material type (lung, liver). In this table, all median and mean values of the lung or liver homogenates are listed, sorted by ascending laser fluence and gelatin content, or section thickness of the lung and liver homogenates of the calibrations. Based on this data, individual, group (sub- and supergroups) and total values (minimum, median, mean, percentile and maximum values) are calculated within the table. In addition, data ranges are also defined, which reduces the complexity of the VBA code used below to generate the box-whisker plots and the overview tables.

## Results and discussion

### ICP-MS

To determine the actual contents of the doped materials produced (gelatin calibration materials, lung- and liver-calibration materials, lung and liver samples), they were liquified using microwave pressure digestion and measured using ICP-MS. In parallel, two NIST standard reference materials were measured for verification. The results are shown in Table 1 (ESI). Unfortunately, the elements As, Cd, Co, Cr, Hg and Ni could not be determined as the concentration in the reference material was too low, also as shown in Table 3. Be, Pt, Y and Zr were not included. However, all other elements were successfully measured.

**Table 3:** Evaluation of lung homogenate samples with gelatin, lung, and liver calibrations

Analyte	C: Gelatin, S: Lung		C: Lung, S: Lung		C: Liver, S: Lung		Reference value
	Median spike	Average spike	Median spike	Average spike	Median spike	Average spike	
	µg/g	µg/g	µg/g	µg/g	µg/g	µg/g	µg/g
9Be	9.96 ± 1.52	18.7 ± 5.26	6.29 ± 2.56	8.06 ± 3.32	14.3 ± 4.94	18.3 ± 6.54	10.2
23Na	11,900 ± 2040	14,300 ± 4260	14,500 ± 4520	14,800 ± 4740	16,500 ± 2540	17,100 ± 3000	11,900
24Mg	1300 ± 152	1680 ± 432	1310 ± 358	1340 ± 374	1920 ± 420	1950 ± 440	1280
27Al	86.4 ± 13.32	131 ± 36.2	54.4 ± 20.8	67.9 ± 25.2	104 ± 31.6	129 ± 39.4	84.5
31P	15,900 ± 2280	20,200 ± 5420	16,700 ± 5960	16,800 ± 6060	24,900 ± 6900	25,100 ± 7060	16,300
39K	18,100 ± 2360	23,500 ± 6240	11,500 ± 3000	11,800 ± 3100	22,800 ± 9260	23,300 ± 9560	18,700
42Ca	916 ± 171.6	1280 ± 380	1010 ± 254	1090 ± 366	1120 ± 318	1230 ± 480	913
44Ca	918 ± 140.8	1210 ± 340	924 ± 262	961 ± 264	926 ± 332	972 ± 338	913
51V	12.6 ± 1.68	18.9 ± 5.04	6.96 ± 1.92	7.60 ± 2.22	12.0 ± 2.48	13.1 ± 3.04	12.6
52Cr	8.00 ± 1.06	10.5 ± 1.88	16.1 ± 6.48	18.1 ± 7.44	15.4 ± 6.76	17.5 ± 7.92	7.70
55Mn	11.5 ± 2.20	15.0 ± 4.48	8.42 ± 2.56	9.02 ± 2.88	26.2 ± 6.14	27.2 ± 6.62	11.2
57Fe	834 ± 135	1280 ± 360	1140 ± 556	1290 ± 640	1620 ± 466	1890 ± 574	829
59Co	8.00 ± 1.70	10.1 ± 2.70	6.58 ± 1.94	7.15 ± 2.42	13.0 ± 2.90	14.1 ± 3.98	8.30
60Ni	15.5 ± 2.22	21.9 ± 5.98	11.3 ± 2.84	12.7 ± 5.78	21.2 ± 4.76	23.9 ± 10.46	15.4
63Cu	14.0 ± 1.34	20.4 ± 3.62	16.9 ± 8.48	18.6 ± 9.58	33.6 ± 8.76	36.4 ± 10.3	13.6
64Zn	125 ± 20.2	175 ± 49.6	110 ± 54.4	118 ± 65.0	188 ± 97.8	204 ± 117.8	127
75As	9.92 ± 1.48	14.9 ± 4.06	7.19 ± 2.10	8.67 ± 4.9	14.7 ± 4.42	17.7 ± 9.94	9.90
89Y	7.05 ± 0.94	10.6 ± 2.86	4.85 ± 2.26	6.56 ± 3.00	8.66 ± 2.52	11.6 ± 3.46	7.20
90Zr	17.0 ± 3.78	56.8 ± 18.4	7.84 ± 5.10	19.8 ± 11.5	14.3 ± 13.2	35.9 ± 31.8	18.1
95Mo	15.6 ± 1.42	21.4 ± 5.40	12.4 ± 2.82	13.5 ± 3.14	23.9 ± 4.82	25.7 ± 5.44	15.6
111Cd	7.75 ± 0.96	10.6 ± 2.78	5.18 ± 2.88	5.96 ± 3.48	9.57 ± 5.16	10.9 ± 6.26	8.30
195Pt	8.00 ± 0.62	10.7 ± 1.42	5.41 ± 2.36	6.83 ± 2.86	8.47 ± 1.74	10.6 ± 2.36	8.10
202Hg	7.11 ± 0.96	9.77 ± 2.64	5.47 ± 2.14	6.19 ± 2.34	9.21 ± 2.50	10.3 ± 2.86	7.20

The table shows the calculated results of the lung homogenate samples of all measured elemental isotopes with evaluation via median and mean value generation, in each case without elemental spike removal. The 2nd and 3rd column (C: Gelatin, S: Lung) show the evaluations with gelatin calibrations with *n* = 5400. The 4th and 5th column (C: Lung, S: Lung) show the evaluations with lung homogenate calibrations (*n* = 1800) and the last two columns (C: Liver, S: Lung) show the evaluations with liver homogenate calibrations (*n* = 1800)

*C* calibration, *S* sample, *SD* standard deviation (2SD)

Table 2a–c (ESI) displays the values found for the doped materials. The gelatin (Table 2a, ESI) showed no detectable element contamination, allowing for direct reading of the contents from the table. However, for lung and liver samples (Table 2b and c, ESI), some of the analyzed elements were already present in the starting material, resulting in an offset for these elements. As a result, it is not possible to directly determine the contents, requiring the use of the standard addition method.

### Calibration

Regarding the gelatin calibrations produced, only the calibrations with 0.7, 2.0, 5.0, 7.0, 8.0 and 9.0% gelatin content could be evaluated. Gelatin content below 0.7% provided too little difference for error-free detecting using automatic section margin detection (Fig. 1, ESI).

Although the gelatin content of 0.7% was accurately recognized, the correlation coefficients (Sect. "Data processing, visualization and statistics") with a mean of 0.9603 show that a higher gelatin content is preferable (2.0% 0.9981 and higher). However, the values from the calibration series with 0.7% were still included in the analyses because good recoveries could be achieved with them. Based on the gelatin calibrations used to assess the sample sections, slight chromatography effects (coffee-ring effect/Marangoni effect) were observed visually. Nevertheless, the recoveries did not provide any justification for excluding the calibrations from the analysis.

In calibrations with a gelatin content higher than 9.0% (15%, 20%), the individual calibration points correlated very well with each other, achieving correlations of 0.9988 and 0.9969. However, the recoveries were worse. Upon visual inspection of the ablated calibration lines, it appears that the gelatin film appears is too thick, and the relatively high concentration of elements (resulting in strong crystallization formation on the surface) makes the film too solid for the laser to pass through completely at the selected fluences. Possible inhomogeneities in the depth of the calibration lines, caused by increased concentrations on the surface of the calibration paths, had a particularly strong effect. As a result, these results were not included in the analyses.

For quality-control purposes and as sample material, doped lung- and liver-homogenized sections were prepared on the cryomicrotome at thicknesses of 10, 20 and 30 µm. It was observed that the lung material (from young bull cattle lungs) was the most challenging to homogenize due to the presence of numerous branched bronchi. Despite this difficulty, there were strong correlations observed within the standard series, with correlations increasing as thickness increased, especially for lung tissue. On average, correlations for lung tissue were 0.9775 for 10 µm 0.9909 for 20 µm, and 0.9922 for 30 µm. For liver tissue, the correlations were 0.9924 for 10 µm, 0.9940 for 20 µm, and 0.9946 for 30 µm. Remarkably, correlations as high as 0.9999 and even 1.000 were also observed for all three materials.

### Samples

Thirty sections each of the fourth calibration standard (S4) of lung and liver tissue were used as samples. The sections were prepared in 10, 20 and 30 µm, with two sections per fluence (0.50, 0.75, 1.00, 1.25, and 1.50 J/cm^2^). All sections were applied to all analyzed calibrations. Due to the large number of calibrations and samples, they could not be measured in one run, so the ICP-MS and LA unit had to be switched off in the meantime. In addition, a liver calibration with a thickness of 30 µm can be applied to samples with a thickness of 10 or 20 µm without any differences observed, presuming that a signal normalization (here carried out via the ^13^C signal) has been performed. Therefore, the results for all fluences and thicknesses were grouped together.

First, we need to consider the different methods of calculating the values. These include the calculation of median and mean values, both with and without de-spiking. This is only done for samples, as removing these signals for calibration could result in inaccurate calculations. The procedure is detailed in Sect. "Data processing, visualization and statistics". It is important to note explicitly that zero values were not taken into account when calculating both variables, as this would significantly skew the results. As expected, the mean values were higher than the median values. Since the medians are already sorted in ascending order and the middle value is the median, de-spiking is inherently included in the median calculations. This explains why there were minimal differences between "de-spike" and "spike" in the median values. However, when it comes to mean values, there is a significant difference. This is due to the fact that when calculating the contents without de-spiking, the intensities of each individual data point of a section that lies within the section margins are transformed unaltered into contents. Even if the intensity is a highly overestimated value ("spike"). While some elements, like ^24^ Mg, ^31^P and ^39^ K, show little variation even with spikes due to their high concentrations, others like ^111^Cd, ^195^Pt and ^202^Hg exhibit minimal changes despite occurring in lower concentrations. ^90^Zr stand out as an extreme example of spike inclination, with de-spiking resulting in almost a 100% difference between the median and mean values, and a staggering 250% difference compared to the mean values without de-spiking.

In addition, the type of material used also plays a role in these differences, with gelatin showing the highest variations for zirconium, at over three times as much. Tables 3 (lung samples) and 4 (liver samples) display all medians and mean values without de-spiking, providing a clearer picture of these discrepancies. The values were calculated for all laser fluences (0.50–1.50 J/cm^2^) and in the case of the gelatin calibrations, for all gelatin concentrations (0.7–9.0%). Similarly, for lung and liver calibrations, the values were calculated for all section thicknesses (10–30 µm). The values were corrected using ^13^C as an internal standard.

Tables 3 and 4 also indicate that the recoveries are heavily influenced by the calibration material used for analysis. When examining the lung samples analyzed with lung calibrations (Table 3, C: Lung, median), it is evident that they are significantly under-recovered. Conversely, when analyzed with liver calibrations (Table 3, C: Liver, median), the recoveries are generally over-recovered. A comparison of the liver samples reveals a different pattern. When analyzed with lung calibrations (Table 4, C: Lung, median), it is apparent that these samples also tend to be under-recovered, but are much closer to the reference values. On the other hand, when liver calibrations (Table 4, C: Liver, median) are used for analysis, the recoveries are mostly over-recovered, yet closer to the reference values compared to the lung samples. Nevertheless, the reference values are consistently met and rarely fall short.

**Table 4:** Evaluation of liver homogenate samples with gelatin, lung and liver calibrations

Analyte	C: Gelatin, S: Liver		C: Lung, S: Liver		C: Liver, S: Liver		Reference value
	Median spike	Average spike	Median spike	Average spike	Median spike	Average spike	
	µg/g	µg/g	µg/g	µg/g	µg/g	µg/g	µg/g
9Be	9.89 ± 1.66	12.4 ± 3.52	5.77 ± 2.26	5.93 ± 2.22	13.4 ± 3.58	13.7 ± 3.66	10.3
23Na	8110 ± 1274	8320 ± 1558	13,000 ± 4080	13,200 ± 4120	14,400 ± 1970	14,600 ± 2000	8140
24Mg	1560 ± 278	1960 ± 578	1740 ± 426	1750 ± 428	2470 ± 474	2480 ± 478	1560
27Al	89.1 ± 16.9	118 ± 35.8	78.6 ± 28.6	74.3 ± 330	149 ± 45.6	141 ± 56.0	85.2
31P	19,200 ± 2920	24,300 ± 6740	17,600 ± 6140	17,700 ± 6140	26,300 ± 6980	26,400 ± 6980	19,700
39K	17,200 ± 2780	22,900 ± 6520	10,100 ± 2760	10,500 ± 2940	19,800 ± 8220	20,600 ± 8660	17,900
42Ca	726 ± 154.6	1020 ± 322	779 ± 202	838 ± 420	809 ± 228	889 ± 548	690
44Ca	704 ± 108	1010 ± 278	650 ± 196	714 ± 512	584 ± 220	664 ± 638	690
51V	12.0 ± 0.44	12.3 ± 0.54	6.17 ± 1.56	6.24 ± 1.60	10.6 ± 1.80	10.8 ± 1.86	12.1
52Cr	8.44 ± 1.72	11.6 ± 3.6	15.9 ± 6.76	17.8 ± 19.2	15.1 ± 7.06	17.1 ± 20.4	8.40
55Mn	23.5 ± 3.94	30.5 ± 8.78	15.5 ± 3.64	16.0 ± 4.00	38.4 ± 8.30	39.3 ± 8.82	24.4
57Fe	833 ± 91.8	1070 ± 274	1060 ± 520	1050 ± 518	1490 ± 426	1470 ± 438	839
59Co	9.00 ± 0.76	9.44 ± 0.98	5.26 ± 1.5	5.33 ± 1.54	10.5 ± 1.96	10.6 ± 2.08	8.70
60Ni	15.0 ± 0.78	30.8 ± 3.74	9.19 ± 2.14	9.84 ± 5.00	17.4 ± 3.34	18.6 ± 8.98	15.4
63Cu	26.0 ± 1.40	26.3 ± 1.86	23.2 ± 11.3	23.4 ± 11.5	44.1 ± 11.1	44.5 ± 11.4	26.3
64Zn	150 ± 15.9	193 ± 49.0	105 ± 47.6	106 ± 49.2	178 ± 85.0	181 ± 87.8	149
75As	10.0 ± 1.18	12.2 ± 2.02	5.36 ± 1.48	5.72 ± 1.68	11.1 ± 3.12	11.8 ± 3.52	10.0
89Y	6.78 ± 0.94	8.49 ± 2.32	5.77 ± 2.68	5.39 ± 2.52	10.3 ± 2.98	9.55 ± 3.04	7.30
90Zr	20.5 ± 6.64	38.1 ± 17.3	6.4 ± 6.88	13.1 ± 10.6	13.2 ± 15.8	25.0 ± 26.8	19.6
95Mo	20.0 ± 1.90	20.6 ± 2.04	12.4 ± 2.86	12.6 ± 2.90	23.7 ± 4.88	24.1 ± 4.98	19.5
111Cd	8.74 ± 1.56	10.9 ± 3.22	4.72 ± 2.76	5.02 ± 3.08	8.8 0 ± 4.84	9.31 ± 5.46	9.30
195Pt	9.00 ± 1.00	9.05 ± 0.90	5.43 ± 2.74	5.46 ± 2.68	8.65 ± 2.20	8.69 ± 2.08	9.40
202Hg	8.82 ± 1.06	11.0 ± 2.86	5.06 ± 2.06	5.12 ± 2.14	8.68 ± 2.20	8.75 ± 2.48	8.70

The table shows the calculated results of the liver homogenate samples of all measured elemental isotopes with evaluation via median and mean value generation, in each case without elemental spike removal. The 2nd and 3rd column (C: Gelatin, S: Lung) show the evaluations with gelatin calibrations with *n* = 5400. The 4th and 5th column (C: Lung, S: Lung) show the evaluations with lung homogenate calibrations (*n* = 1800) and the last two columns (C: Liver, S: Lung) show the evaluations with liver homogenate calibrations (*n* = 1800)

*C* calibration, *S* sample, *SD* standard deviation (2SD)

If, gelatin is used for the evaluation and the median values are selected instead, the reference values are either hit directly or very close to them. This applies to both lung and liver samples.

**Fig. 2:**
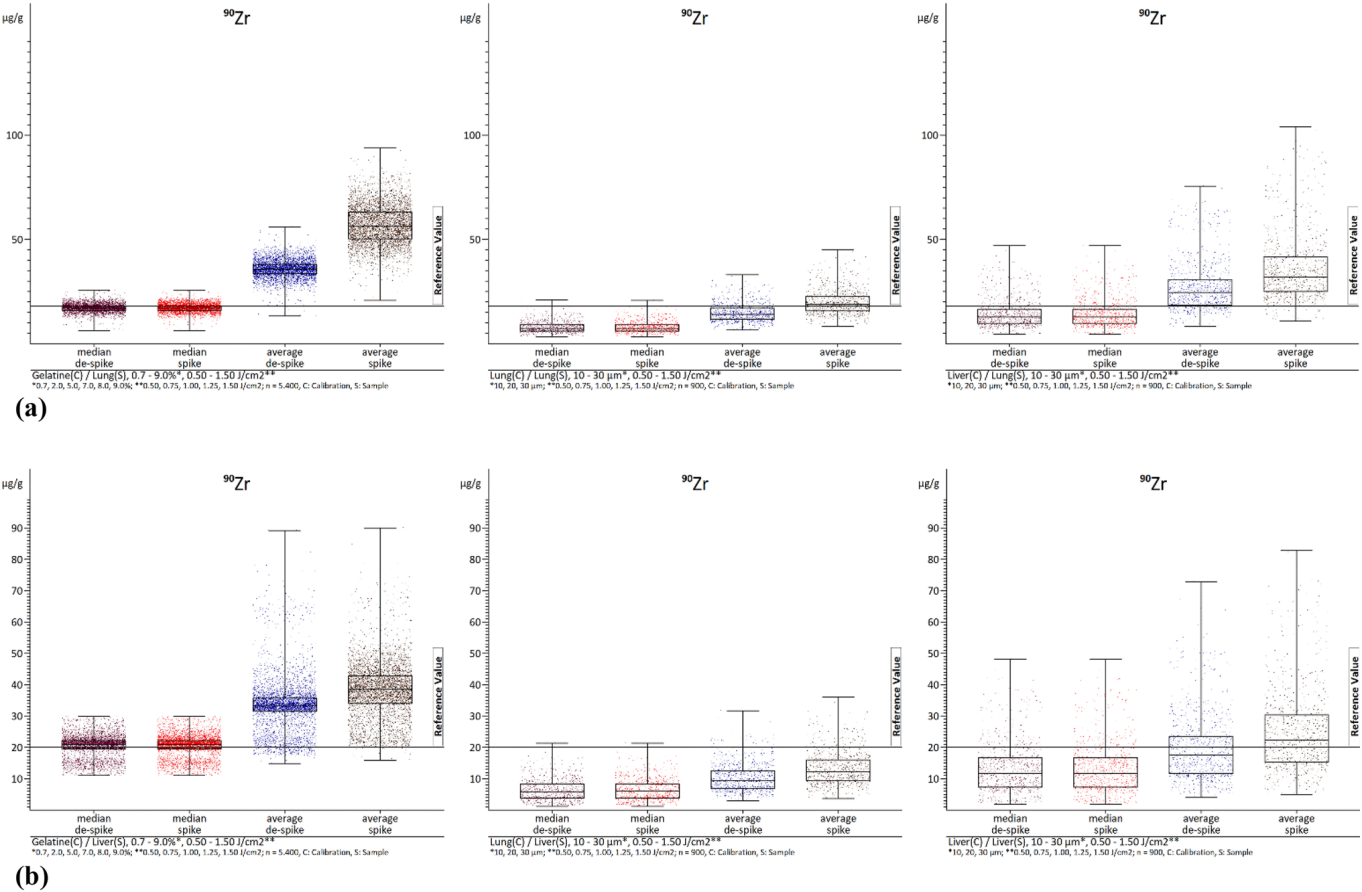
**a** The images show box-whisker plots of ^90^Zr. The median and average with and without de-spiking (elemental spike) of the lung homogenate samples are visualized. The left image displays the evaluations with gelatin calibrations with *n* = 5400. The middle image represents the evaluations with lung homogenate calibrations with *n* = 1800. The right image illustrates the evaluations with liver homogenate calibrations with *n* = 1800. *C* calibration, *S* sample. **b** The images show box-whisker plots of ^90^Zr. The median and average with and without de-spiking (elemental spike) of the liver homogenate samples are visualized. The left image displays the evaluations with gelatin calibrations with *n* = 5400. The middle image represents the evaluations with lung homogenate calibrations with *n* = 1800. The right image illustrates the evaluations with liver homogenate calibrations with *n* = 1800. *C* calibration, *S* sample

**Fig. 3:**
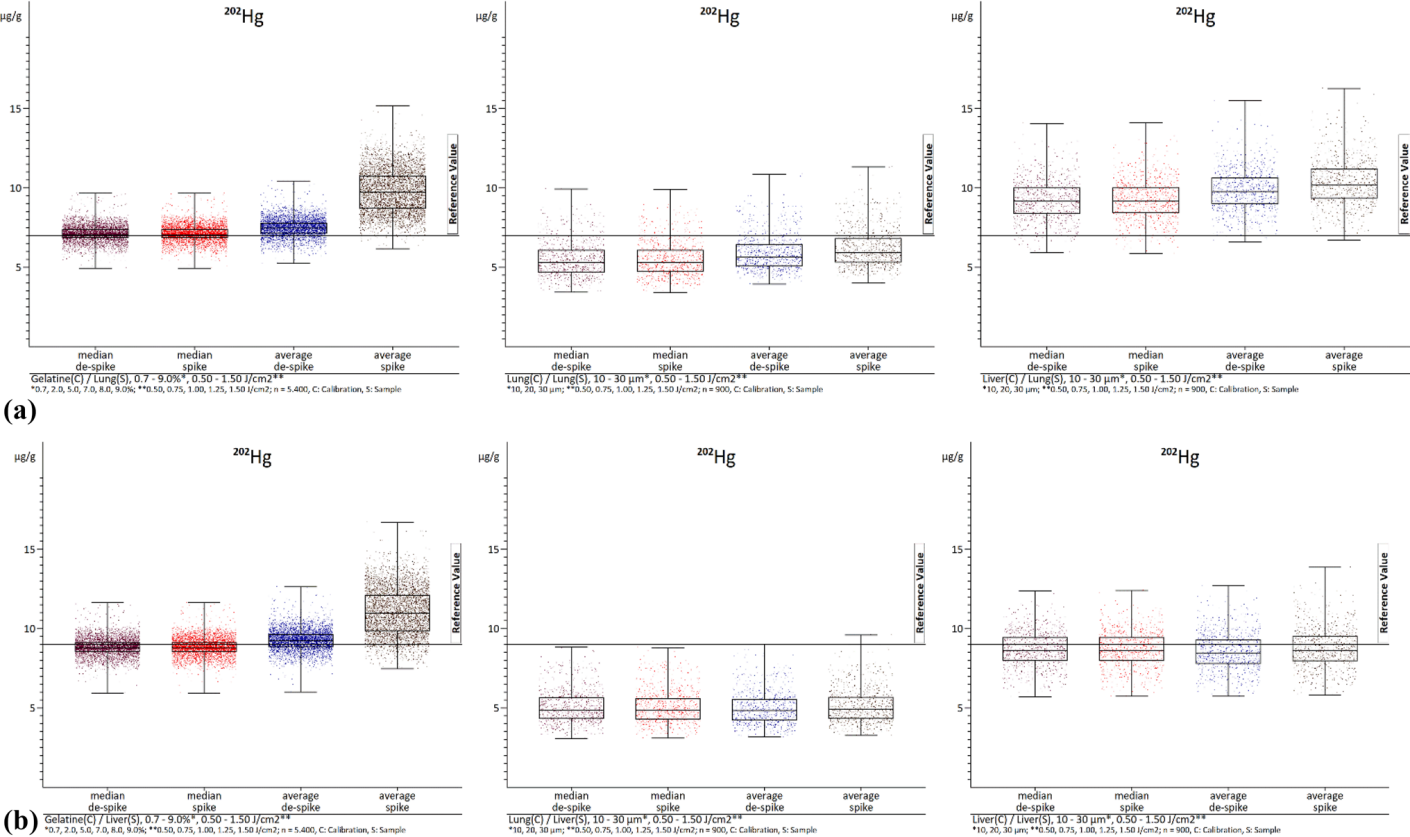
**a** The images show box-whisker plots of ^202^Hg. The median and average with and without de-spiking (elemental spike) of the lung homogenate samples are visualized. The left image displays the evaluations with gelatin calibrations with *n* = 5400. The middle image represents the evaluations with lung homogenate calibrations with *n* = 1800. The right image illustrates the evaluations with liver homogenate calibrations with *n* = 1800. *C* calibration, *S* sample. **b** The images show box-whisker plots of ^202^Hg. The median and average values with and without de-spiking (elemental spike) of the liver homogenate samples are visualized. The left image shows the evaluations with gelatin calibrations with *n* = 5400. The middle image represents the evaluations with lung homogenate calibrations with *n* = 1800. The right image illustrates the evaluations with liver homogenate calibrations with *n* = 1800. *C* calibration, *S* sample

Figures 2a, b, 3a, and b show examples of the absolute distributions of measured values of ^90^Zr (as an example with extreme spikes) and ^202^Hg (as an example with few spikes) in box-whisker plots, with evaluations of lung (Figs. 2a, 3a) and liver (Figs. 2b, 3b) samples in each case. The values represent summaries of all laser fluences (0.50–1.50 J/cm^2^). In the case of the gelatin calibrations, all gelatin concentrations (0.7–9.0%) were combined, while for the lung and liver calibrations, all section thicknesses (10–30 µm) were included. The internal standard used was ^13^C. While other analyzed elements are not depicted (see ESI), they exhibit similar distribution patterns. The diagrams illustrate that, as previously mentioned, lung samples are under-recovered, and liver samples are over-recovered. Moreover, this type of diagrams provides a clearer visualization of the impact of de-spiking and the distribution of individual measured values. It is evident that calibrations with gelatin (left) result in more evenly and closely distributed values.

In contrast, analyses using lung (middle) and liver (right) tissue calibrations can result in more extensive distributions. Specifically, the mean values often shift the center (50th percentile) of the values without de-spiking. Especially at ^90^Zr in the representation of the liver homogenate samples (Fig. 2b), de-spiking appears to have little effect on the mean values. This is because there are so many elementary spikes occur that they still strongly influence the mean values even with the method used for spike removal. The value distributions for the gelatin calibrations also appear particularly strong, but this is due to the high under-recovery of the liver homogenate calibrations. The distribution is actually best in the evaluations with the gelatin calibrations. However, it can be seen that the spike removal has an effect in the other images (Figs. 2a, 3a and b). When de-spiking and selecting medians, the range of values decreases. It is evident that the range of value distributions, especially in the median range, is largest in the analyses with liver calibrations, followed by lung and gelatin calibrations. Based on current knowledge, the gelatin calibrations showing a smaller range of values is mainly due to the ease of homogenizing gelatin element mix compared to the liver liver/lung element mixes. Surprisingly, the lung element mix appears more homogeneous than the liver element mix, despite the lung tissue being more difficult to homogenize. In addition, when using tissue homogenizes for calibrations, a significantly smaller area of the section is ablated compared to using it as a sample where the entire section is ablated. This means that possible inhomogeneities have a greater impact, when using tissue homogenates for calibration, as opposed to being leveled out by complete ablation when used as samples. This is evident in the box-whisker plots of the gelatin analyses. Any differences in the presentation of values (box-whisker plot) may be due to chromatography effects, but overall, they do not affect the precision of the complete evaluation. However, there is still potential for optimization to further enhance this simple universal method of calibration production within LA-ICP-MS.

In the experiments conducted here, we selected conditions commonly used in our laboratories for analyzing tissue sections. This included choosing specific section thicknesses for the tissue sections and determining the necessary laser fluences. The higher fluences (1.25/1.50 J/cm^2^) proved to be adequate for completely ablating all tissues types and thicknesses without significantly affecting the underlying glass surface. However, at higher fluences, there is a risk of damaging the glass surface and thus causing coablation with unwanted contributions from elements like Ca, Na, Mg or K. Lower laser fluences, such as 0.50 and 0.75 J/cm^2^, and occasionally 1.00 J/cm^2^, resulted in incomplete ablations, particularly with thicker tissue section. This issue was not significant in lung and liver samples due to their high degree of homogenization, but could lead to inaccurate measurements in real samples. Therefore, we determined that a laser fluence between 1.25 and 1.50 J/cm^2^ is ideal. We were unable to replicate changes in element ratios analyte/13C described by Makino and Nakzako [32] in our experiments. The discrepancies may be due to differences in experimental designs and settings, such as the LA-Unit, ICP-MS-Device, spot size, sample type, and laser fluence used. Despite this, the correcting measured values in biologic samples using 13C as an internal standard is a well-established method, and our results support its validity.

As previously mentioned in Sect. "Samples", the ICP-MS could not be operated continuously due to the large number of measurements required for these experiments. The measurements were therefore conducted over seven sessions, totaling approximately 50 days. Due to the significant amount of data generated, it is not feasible to present a graphical representation of all individual results in this work or in the ESI. However, this information can be provided upon request. Surprisingly, no differences in laser fluence, section thickness, or gelatin content were observed during these experiments. Furthermore, no negative effects of the intermittent ICP-MS operation were detected.

**Fig. 4:**
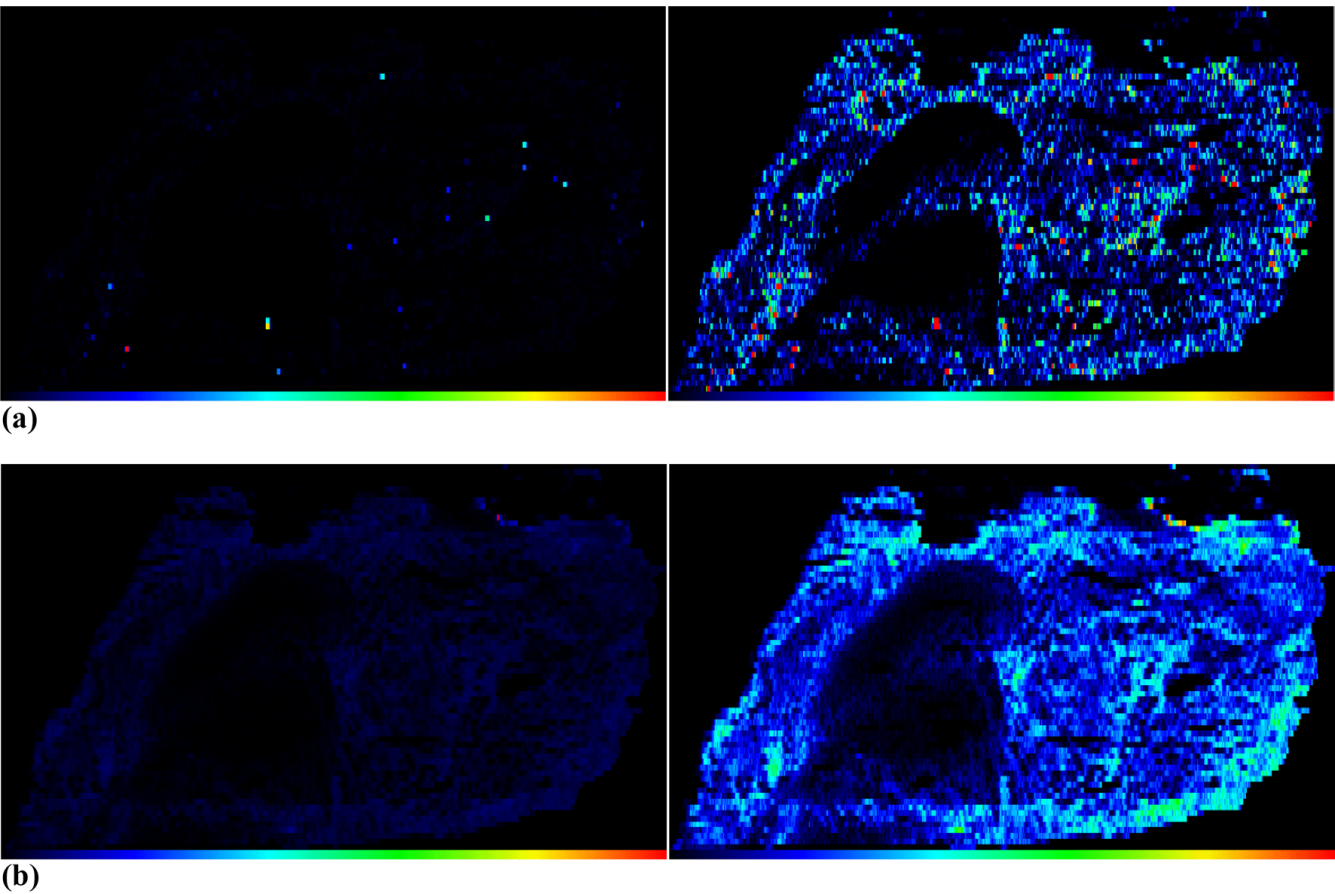
**a** The images in this figure show computer-assisted image generation of a lung homogenate sample with a section thickness of 30 µm, which was ablated with a laser fluence of 1.00 J/cm^2^. The images show the intensity distribution of the isotope 90Zr. The left image shows the image without elementary spike removal, the right image features de-spiking and more details are visible. **b** The images in this figure show computer-assisted image generation of a lung homogenate sample with a section thickness of 30 µm, which was ablated with a laser fluence of 1.00 J/cm2. The images show the intensity distribution of the isotope ^202^Hg. The left image shows the image without elementary spike removal, the right-image features de-spiking and more details are visible

However, the method for calculating median and mean values is only relevant if, as in this study, it is necessary to verify whether the calibrations work in principle, i.e., whether the median or mean value is to be formed over the entire section. According to the available results, no de-spiking is necessary if the median is selected. However, if images are to be generated, computer- assisted de-spiking is required. This is because our imaging software typically creates a color scale from zero to the maximum value in order to accurately display all intensities or contents. If the data set contains elementary spikes that have not been removed, the generated images appear disproportionately dark (Fig. 4a, b, left) and it is difficult to identify details. By removing these elementary spikes with following image generation, the images can be visualized better (Fig. 4a, b, right) without removing important information from the images. These images finally also show that elemental peaks occur in the tissue homogenate samples (lung, liver), more in the case of ^90^Zr than in the case of ^202^Hg.

## Conclusions

In conclusion, gelatin with a content of between 2 and 9% is highly suitable as a calibration matrix. It is easy to prepare, can be pipetted onto an object slide and then dried in the air. Since no cryomicrotome is needed for the calibrations themselves, only for the sample sections, this method also saves a significant amount of time. These experiments have shown that calibration using tissue homogenates in the conventional way is associated with many uncertainties. These uncertainties include increased homogenization effort, sectioning on the microtome and accurate positioning of the calibration sections on the slide.

Additional insights from the experiments show that the reference values determined using microwave-assisted ICP-MS are best obtained with gelatin as the calibration material. In addition, the results revealed an unexpected observation that the influences of laser fluences, gelatin contents, and section thicknesses are relatively minor. Interruptions in the measurement process between samples and calibrations also had no measurable effects on the results. Therefore, it is possible to separate calibrations and samples, even with interruptions, at least under the conditions used in this study. The optimal range for selecting laser fluences was found to be between 1.25 and 1.50 J/cm^2^.

## Supplementary Information

Below is the link to the electronic supplementary material.

Supplementary file1 (DOCX 38222 KB)

## Data Availability

The data supporting the findings of this study are available within the paper. Data will be made available on reasonable request.
