# Supplementary material for: Line-dropped gelatin multi-element calibration standards in LA-ICP-MS: a statistically verifying comparison with cryosectioned homogenized lung and liver as matrix-matched calibration standards and as corresponding reference materials
Source: Anal Sci. 2024 Nov 27;41(3):237–49. doi: 10.1007/s44211-024-00691-8 (PMC11832574; doi:10.1007/s44211-024-00691-8)

*Supplements*

Line-dropped gelatin multi-element calibration standards in LA-ICP-MS: A statistically verifying comparison with cryosectioned homogenised lung and liver as matrix-matched calibration standards and as corresponding reference materials

Sven Thoröe-Boveleth ^a^, Ruth Becker ^a^, Jens Bertram ^a^, Thomas Schettgen ^a^, Manfred Möller ^a^, Danny Jonigk ^c, d^, Thomas Kraus ^a,†^ and Ralf Weiskirchen ^b,†^

**Supplementary Tables**

ICP-MS measurement of two NIST SRM´s for quality control

| Bovine liver, NIST SRM 1577C | | | **Table 1** Determination of two NIST Standard Reference Materials with specification of standard deviation and reference values (n = 3). | Whole Milk Powder, NIST SRM 1549a | | |
| --- | --- | --- | --- | --- | --- | --- |
| Sample name | measured | certified reference values |  | Sample name | measured | certified reference values |
| Analyte | µg/g | µg/g |  | Analyte | µg/g | µg/g |
| Al | - | - |  | Al | 1.98 ± 0.41 | 2* |
| As | < 0.50 | 0.0196 ± 0.0014 |  | As | < 0.50 | 0.0019* |
| Be | - | - |  | Be | - | - |
| Ca | 132 ± 2.25 | 131 ± 10.0 |  | Ca | 12,900 ± 251 | 13,000 ± 500 |
| Cd | < 0.50 | 0.097 ± 0.0014 |  | Cd | < 0.50 | 0.0005 ± 0.0002 |
| Co | < 0.50 | 0.3 ± 0.0018 |  | Co | < 0.50 | 0.0041* |
| Cr | < 0.50 | 0.053 ± 0.0014 |  | Cr | < 0.50 | 0.0026 ± 0.0007 |
| Cu | 273 ± 71.1 | 275 ± 4.60 |  | Cu | 0.70 ± 0.21 | 0.70 ± 0.10 |
| Fe | 199 ± 53.9 | 198 ± 0.65 |  | Fe | 1.80 ± 0.35 | 1.78 ± 0.10 |
| Hg | - | - |  | Hg | < 0.50 | 0.0003 ± 0.0002 |
| K | 10,200 ± 297 | 10,200 ± 640 |  | K | 16,800 ± 503 | 16,900 ± 300 |
| Mg | 622 ± 14.4 | 620 ± 42.0 |  | Mg | 1,200 ± 29.9 | 1,200 ± 30.0 |
| Mn | 10.5 ± 3.04 | 10.5 ± 0.47 |  | Mn | < 0.50 | 0.26 ± 0.06 |
| Mo | 3.28 ± 0.75 | 3.30 ± 0.13 |  | Mo | < 0.50 | 0.34* |
| Na | 2,040 ± 54.8 | 2,030 ± 640 |  | Na | 4,950 ± 119 | 4,970 ± 100 |
| Ni | < 0.50 | 0.0445 ± 0.0092 |  | Ni | - | - |
| P | - | - |  | P | 10,700 ± 207 | 10,600 ± 200 |
| Pt | - | - |  | Pt | - | - |
| V | < 0.50 | 0.00817 ± 0.00066 |  | V | - | - |
| Y | - | - |  | Y | - | - |
| Zn | 183 ± 2.92 | 181 ± 1.00 |  | Zn | 46.2 ± 0.92 | 46.1 ± 2.20 |
| Zr | - | - |  | Zr | - | - |
|  |  |  |  | * contained but not certified | | |

ICP-MS measurement of 8 calibration points (S0 - S7) of gelatin calibration material (non-doped/doped)

| Gelatin | | | | | | | | |
| --- | --- | --- | --- | --- | --- | --- | --- | --- |
| Sample name | S0 | S1 | S2 | S3 | S4 | S5 | S6 | S7 |
| Analyte | µg/g | µg/g | µg/g | µg/g | µg/g | µg/g | µg/g | µg/g |
| Al | n.d. | 9.86 ± 0.50 | 19.7 ± 0.86 | 49.3 ± 2.76 | 78.9 ± 3.72 | 98.6 ± 3.36 | 197 ± 8.16 | 296 ± 16.9 |
| As | n.d. | 0.99 ± 0.04 | 1.98 ± 0.10 | 4.95 ± 0.22 | 7.92 ± 0.34 | 9.90 ± 0.30 | 19.8 ± 0.74 | 29.7 ± 1.48 |
| Be | n.d. | 0.886 ± 0.04 | 1.77 ± 0.08 | 4.43 ± 0.20 | 7.09 ± 0.32 | 8.86 ± 0.32 | 17.7 ± 0.98 | 26.6 ± 1.28 |
| Ca | n.d. | 95.8 ± 5.46 | 192 ± 8.96 | 479 ± 14.8 | 766 ± 39.4 | 958 ± 46.0 | 1,920 ± 58.4 | 2,870 ± 146 |
| Cd | n.d. | 1.00 ± 0.06 | 2.01 ± 0.12 | 5.01 ± 0.20 | 8.02 ± 0.30 | 10.0 ± 0.42 | 20.1 ± 1.18 | 30.1 ± 1.26 |
| Co | n.d. | 0.996 ± 0.04 | 1.99 ± 0.12 | 4.98 ± 0.22 | 7.97 ± 0.40 | 9.96 ± 0.44 | 19.9 ± 1.02 | 29.9 ± 1.62 |
| Cr | n.d. | 0.906 ± 0.06 | 1.81 ± 0.06 | 4.53 ± 0.18 | 7.25 ± 0.26 | 9.06 ± 0.50 | 18.1 ± 0.62 | 27.2 ± 0.92 |
| Cu | n.d. | 0.983 ± 0.04 | 1.97 ± 0.08 | 4.91 ± 0.22 | 7.86 ± 0.38 | 9.83 ± 0.50 | 19.7 ± 0.80 | 29.5 ± 0.98 |
| Fe | n.d. | 99.5 ± 5.64 | 199 ± 10.1 | 498 ± 15.8 | 796 ± 33.4 | 995 ± 49.6 | 1,990 ± 119 | 2,990 ± 107 |
| Hg | n.d. | 1.00 ± 0.04 | 2.00 ± 0.12 | 5.00 ± 0.20 | 8.00 ± 0.48 | 10.0 ± 0.56 | 20.0 ± 1.16 | 30.0 ± 1.24 |
| K | n.d. | 996 ± 49.8 | 1,990 ± 101 | 4,980 ± 220 | 7,970 ± 288 | 9,960 ± 400 | 19,900 ± 856 | 29,900 ± 1,780 |
| Mg | n.d. | 99.4 ± 3.76 | 199 ± 11.8 | 497 ± 28.4 | 795 ± 29.4 | 994 ± 54.6 | 1,990 ± 80.8 | 2,980 ± 99.8 |
| Mn | n.d. | 1.04 ± 0.06 | 2.08 ± 0.08 | 5.20 ± 0.28 | 8.32 ± 0.40 | 10.4 ± 0.52 | 20.8 ± 0.62 | 31.2 ± 1.76 |
| Mo | n.d. | 2.03 ± 0.08 | 4.05 ± 0.18 | 10.1 ± 0.44 | 16.2 ± 0.94 | 20.3 ± 0.84 | 40.5 ± 1.54 | 60.8 ± 3.42 |
| Na | n.d. | 1,010 ± 48.0 | 2,010 ± 84.0 | 5,030 ± 232 | 8,050 ± 464 | 10,100 ± 592 | 20,100 ± 1,060 | 30,200 ± 936 |
| Ni | n.d. | 2.05 ± 0.08 | 4.10 ± 0.22 | 10.3 ± 0.54 | 16.4 ± 0.98 | 20.5 ± 0.80 | 41.0 ± 1.62 | 61.5 ± 3.06 |
| P | n.d. | 1,020 ± 31.8 | 2,030 ± 122 | 5,080 ± 204 | 8,130 ± 332 | 10,200 ± 388 | 20,300 ± 716 | 30,500 ± 1,750 |
| Pt | n.d. | 1.02 ± 0.04 | 2.04 ± 0.10 | 5.11 ± 0.22 | 8.17 ± 0.26 | 10.2 ± 0.44 | 20.4 ± 0.84 | 30.6 ± 1.54 |
| V | n.d. | 1.00 ± 0.06 | 2.00 ± 0.10 | 5.01 ± 0.20 | 8.01 ± 0.38 | 10.0 ± 0.54 | 20.0 ± 0.84 | 30.0 ± 1.20 |
| Y | n.d. | 1.00 ± 0.04 | 2.01 ± 0.12 | 5.01 ± 0.24 | 8.02 ± 0.46 | 10.0 ± 0.30 | 20.1 ± 1.12 | 30.1 ± 0.96 |
| Zn | n.d. | 9.59 ± 0.54 | 19.2 ± 1.14 | 47.9 ± 1.82 | 76.7 ± 2.38 | 95.9 ± 4.32 | 192 ± 6.72 | 288 ± 11.1 |
| Zr | n.d. | 1.85 ± 0.06 | 3.70 ± 0.20 | 9.25 ± 0.28 | 14.8 ± 0.66 | 18.5 ± 0.62 | 37.0 ± 1.54 | 55.5 ± 1.84 |
|  | n.d.: not detectable | |  |  |  |  |  |  |

**Table 2a** Determination of the individual gelatin calibration standards (5.0% gelatin content) with indication of the standard deviation (2SD, n = 3).

ICP-MS measurement of 6 calibration points (S0 – S5) of lung homogenate material (non-doped/doped)

| Lung | | | | | | |
| --- | --- | --- | --- | --- | --- | --- |
| Sample name | S0 | S1 | S2 | S3 | S4 | S5 |
| Analyte | µg/g | µg/g | µg/g | µg/g | µg/g | µg/g |
| Al | 1.7 ± 0.06 | 12.1 ± 0.54 | 22.4 ± 1.22 | 53.5 ± 1.96 | 84.5 ± 3.52 | 105 ± 5.90 |
| As | n.d. | 1.33 ± 0.04 | 2.55 ± 0.12 | 6.23 ± 0.36 | 9.90 ± 0.46 | 12.4 ± 0.52 |
| Be | n.d. | 1.28 ± 0.04 | 2.55 ± 0.12 | 6.38 ± 0.24 | 10.2 ± 0.60 | 12.8 ± 0.58 |
| Ca | 423 ± 22.4 | 484 ± 15.5 | 545 ± 19.1 | 729 ± 42.6 | 913 ± 27.6 | 1,040 ± 52.2 |
| Cd | n.d. | 1.04 ± 0.04 | 2.08 ± 0.06 | 5.19 ± 0.18 | 8.30 ± 0.46 | 10.4 ± 0.42 |
| Co | n.d. | 1.13 ± 0.04 | 2.15 ± 0.10 | 5.23 ± 0.22 | 8.30 ± 0.32 | 10.4 ± 0.34 |
| Cr | n.d. | 1.05 ± 0.04 | 2.00 ± 0.10 | 4.85 ± 0.28 | 7.70 ± 0.28 | 9.60 ± 0.46 |
| Cu | 6.6 ± 0.4 | 7.48 ± 0.28 | 8.35 ± 0.26 | 11.0 ± 0.54 | 13.6 ± 0.42 | 15.4 ± 0.82 |
| Fe | 458 ± 19.6 | 504 ± 16.2 | 551 ± 31.6 | 690 ± 37.2 | 829 ± 49.0 | 922 ± 39.4 |
| Hg | n.d. | 0.90 ± 0.04 | 1.80 ± 0.08 | 4.50 ± 0.16 | 7.20 ± 0.24 | 9.00 ± 0.30 |
| K | 10,300 ± 318 | 11,400 ± 584 | 12,400 ± 602 | 15,600 ± 562 | 18,700 ± 626 | 20,800 ± 676 |
| Mg | 616 ± 32.8 | 699 ± 27.0 | 782 ± 46.6 | 1,030 ± 44.2 | 1,280 ± 60.2 | 1,450 ± 49.2 |
| Mn | 0.70 ± 0.04 | 2.01 ± 0.08 | 3.33 ± 0.16 | 7.26 ± 0.40 | 11.2 ± 0.34 | 13.8 ± 0.56 |
| Mo | 1.20 ± 0.04 | 3.00 ± 0.10 | 4.80 ± 0.20 | 10.2 ± 0.38 | 15.6 ± 0.56 | 19.2 ± 0.92 |
| Na | 6,510 ± 334 | 7,190 ± 374 | 7,860 ± 342 | 9,880 ± 548 | 11,900 ± 562 | 13,200 ± 578 |
| Ni | n.d. | 2.01 ± 0.12 | 3.93 ± 0.12 | 9.66 ± 0.42 | 15.4 ± 0.90 | 19.2 ± 0.72 |
| P | 10,800 ± 592 | 11,400 ± 656 | 12,100 ± 608 | 14,200 ± 802 | 16,300 ± 554 | 17,700 ± 902 |
| Pt | n.d. | 1.01 ± 0.04 | 2.03 ± 0.08 | 5.06 ± 0.18 | 8.10 ± 0.26 | 10.1 ± 0.38 |
| V | 2.90 ± 0.18 | 4.11 ± 0.16 | 5.33 ± 0.18 | 8.96 ± 0.38 | 12.6 ± 0.42 | 15.0 ± 0.68 |
| Y | n.d. | 0.90 ± 0.04 | 1.80 ± 0.06 | 4.50 ± 0.20 | 7.20 ± 0.30 | 9.00 ± 0.30 |
| Zn | 95.0 ± 4.04 | 99.0 ± 3.90 | 103 ± 4.06 | 115 ± 5.52 | 127 ± 6.72 | 135 ± 5.56 |
| Zr | 4.40 ± 0.18 | 6.11 ± 0.26 | 7.83 ± 0.40 | 13.0 ± 0.54 | 18.1 ± 1.00 | 21.5 ± 0.76 |
|  | n.d.: not detectable | |  |  |  |  |

**Table 2b** Determination of the individual lung calibration standards with indication of the standard deviation (2SD, n = 3).

ICP-MS measurement of 6 calibration points (S0 – S5) of liver homogenate material (non-doped/doped)

| Liver | | | | | | |
| --- | --- | --- | --- | --- | --- | --- |
| Sample name | S0 | S1 | S2 | S3 | S4 | S5 |
| Analyte | µg/g | µg/g | µg/g | µg/g | µg/g | µg/g |
| Al | n.d. | 10.7 ± 0.36 | 21.3 ± 1.10 | 53.3 ± 1.64 | 85.2 ± 4.58 | 107 ± 6.20 |
| As | n.d. | 1.43 ± 0.06 | 2.65 ± 0.12 | 6.33 ± 0.20 | 10.0 ± 0.48 | 12.5 ± 0.58 |
| Be | n.d. | 1.29 ± 0.08 | 2.58 ± 0.10 | 6.44 ± 0.32 | 10.3 ± 0.56 | 12.9 ± 0.44 |
| Ca | 226 ± 13.26 | 284 ± 14.3 | 342 ± 13.0 | 516 ± 16.5 | 690 ± 25.2 | 806 ± 41.4 |
| Cd | 0.20 ± 0.02 | 1.34 ± 0.06 | 2.48 ± 0.14 | 5.89 ± 0.28 | 9.30 ± 0.50 | 11.6 ± 0.66 |
| Co | n.d. | 1.18 ± 0.04 | 2.25 ± 0.10 | 5.48 ± 0.26 | 8.70 ± 0.28 | 10.9 ± 0.64 |
| Cr | n.d. | 1.14 ± 0.06 | 2.18 ± 0.10 | 5.29 ± 0.22 | 8.40 ± 0.42 | 10.5 ± 0.42 |
| Cu | 17.5 ± 0.72 | 18.6 ± 0.78 | 19.7 ± 1.12 | 23.0 ± 1.32 | 26.3 ± 1.12 | 28.5 ± 1.64 |
| Fe | 358 ± 17.26 | 418 ± 14.72 | 478 ± 23.2 | 658 ± 24.8 | 839 ± 33.8 | 959 ± 37.4 |
| Hg | 2.00 ± 0.10 | 2.84 ± 0.10 | 3.68 ± 0.22 | 6.19 ± 0.20 | 8.70 ± 0.30 | 10.4 ± 0.44 |
| K | 9,300 ± 344 | 10,400 ± 546 | 11,400 ± 670 | 14,700 ± 822 | 17,900 ± 978 | 20,100 ± 736 |
| Mg | 846 ± 44.6 | 935 ± 52.2 | 1,020 ± 53.0 | 1,290 ± 41.6 | 1,560 ± 56.6 | 1,740 ± 79.2 |
| Mn | 13.5 ± 0.76 | 14.9 ± 0.58 | 16.2 ± 0.54 | 20.3 ± 1.16 | 24.4 ± 0.74 | 27.1 ± 1.20 |
| Mo | 3.60 ± 0.20 | 5.59 ± 0.28 | 7.58 ± 0.28 | 13.5 ± 0.78 | 19.5 ± 0.70 | 23.5 ± 1.36 |
| Na | 2,550 ± 85.4 | 3,250 ± 98.8 | 3,950 ± 236 | 6,040 ± 352 | 8,140 ± 476 | 9,540 ± 354 |
| Ni | n.d. | 1.93 ± 0.08 | 3.85 ± 0.20 | 9.63 ± 0.44 | 15.4 ± 0.92 | 19.3 ± 0.66 |
| P | 12,500 ± 692 | 13,400 ± 528 | 14,300 ± 770 | 17,000 ± 730 | 19,700 ± 1,170 | 21,500 ± 776 |
| Pt | n.d. | 1.18 ± 0.04 | 2.35 ± 0.10 | 5.88 ± 0.18 | 9.40 ± 0.50 | 11.8 ± 0.46 |
| V | 3.50 ± 0.14 | 4.58 ± 0.22 | 5.65 ± 0.22 | 8.88 ± 0.50 | 12.1 ± 0.66 | 14.3 ± 0.68 |
| Y | n.d. | 0.913 ± 0.04 | 1.83 ± 0.08 | 4.56 ± 0.24 | 7.30 ± 0.34 | 9.13 ± 0.44 |
| Zn | 106 ± 6.06 | 111 ± 5.84 | 117 ± 6.22 | 133 ± 5.36 | 149 ± 4.90 | 160 ± 8.94 |
| Zr | n.d. | 2.45 ± 0.14 | 4.90 ± 0.20 | 12.3 ± 0.46 | 19.6 ± 0.80 | 24.5 ± 0.84 |
|  | n.d.: not detectable | |  |  |  |  |

**Table 2c** Determination of the individual liver calibration standards with indication of the standard deviation (2SD, n = 3).

Imaging of ablated calibrations with gelatin and tissue homogenates (lung, liver)

**Fig. 1** The figure illustrates the principle of section margin detection via VBA code. As standard practice, a calibration consists of five ablation lines. Within these lines, green indicates low intensities, while yellow and red indicate higher and very high intensities, respectively. The vertical bars (pink, green, white) represent the distinction between background signals and positive signals. White indicates background signals, pink indicates positive signals, and green represents a "blurring" of clusters of pink-colored fields, as continuous bars are required for accurate section margin detection.

First, the sum values of the intensities of all isotopes in each ablation line of a calibration - except for 23Na (which has an ubiquitous distribution) - are calculated for each measured time point, and the minimum of all sum values is determined. A positive signal (pink) is obtained if a sum value exceeds the minimum sum value by more than 10%.

Illustration 1 shows the section margin detection for a blank (the first section of the calibration) from a functioning gelatin calibration. The complete calibration can be seen on the left. The pink and green bars on the right side of the image clearly demonstrate that the section margin detection still encounters issues in the edge area; differences between background signals and positive signals remain small, with considerable background present outside the section. However, this is sufficient for adequate detection.

The second image displays the section margin detection of the second section (first standard) from a functioning liver homogenate calibration on the left. Figure 3 presents the section margin detection for a blank from a functioning lung homogenate calibration, while Figure 4 illustrates one instance from the fifth section (fourth standard) of a lung homogenate calibration where section margin detection failed due to excessive cracks occurring in the section when it was applied to the slide.

**
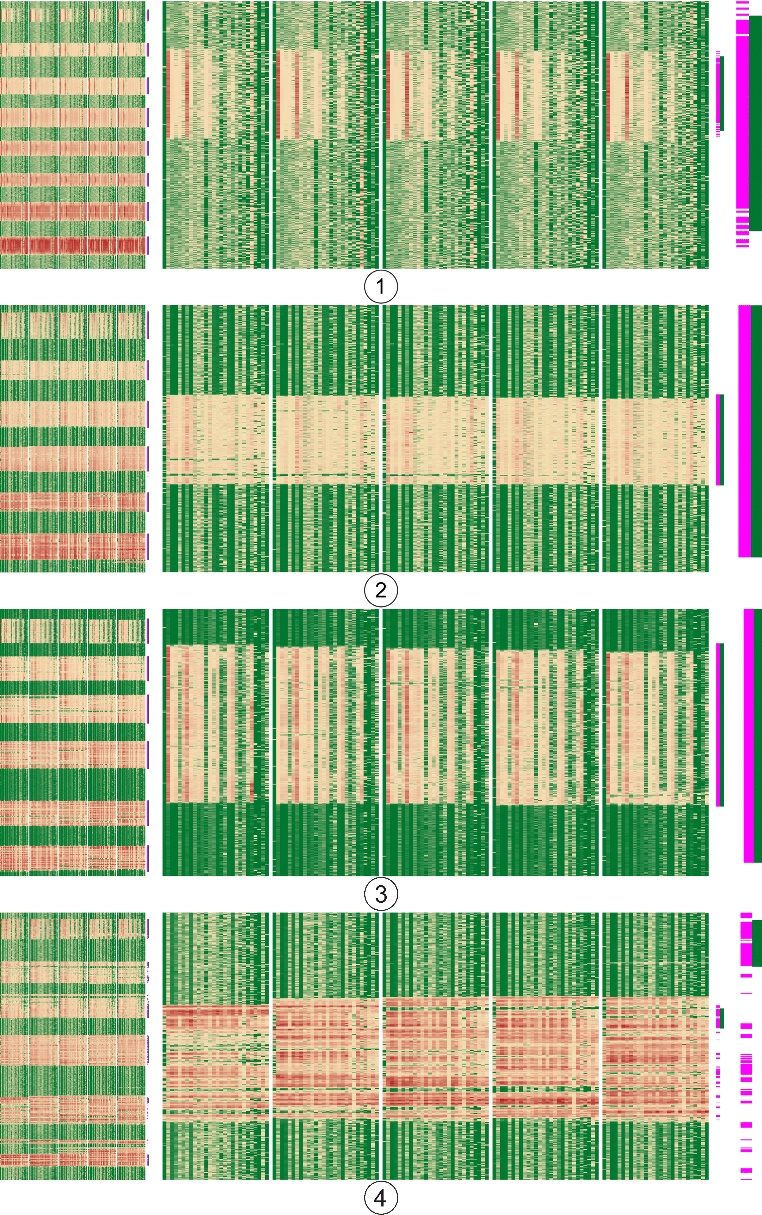
**

Table of the correlation coefficients for gelatin calibrations (0.7%, 2.0%)


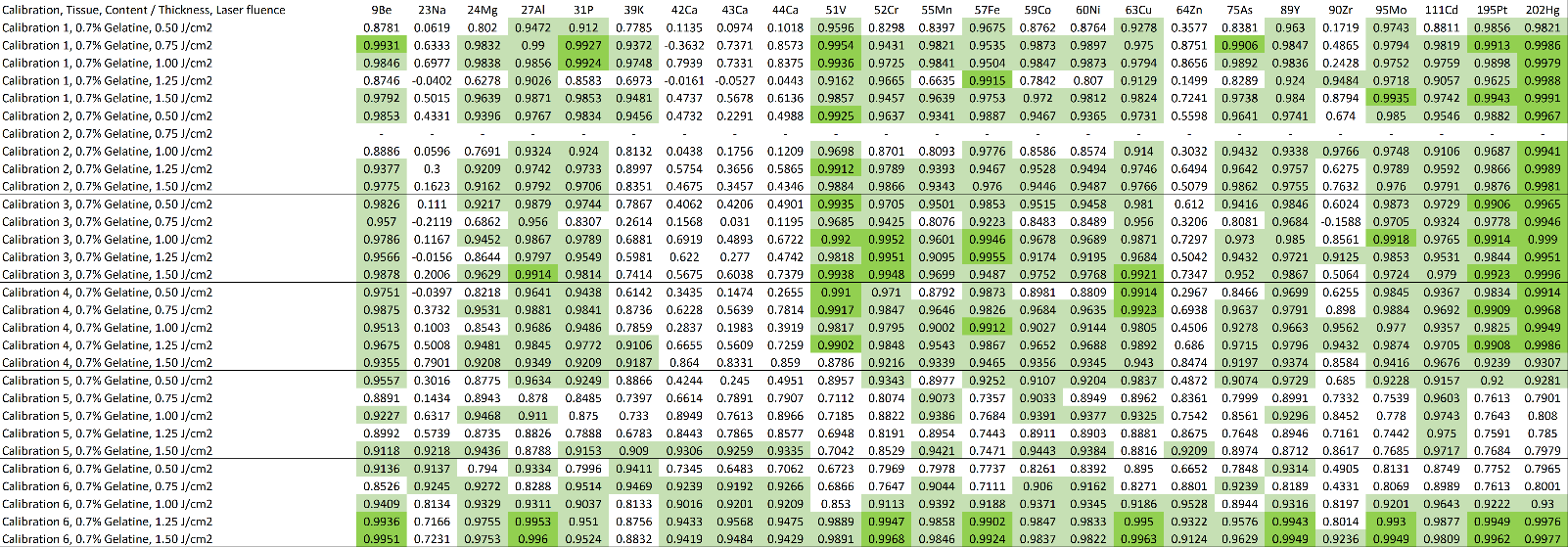


**Table 3a.** Depicted are six calibrations, each with ablation energies of 0.50, 0.75, 1.00, 1.25 and 1.50 J/cm2. Light green indicates a correlation coefficient equal to or greater than 0.9, while darker green signifies a correlation coefficient better than or equal to 0.99. The top table shows the correlation coefficients of gelatin concentrations with 0.7% gelatin content, while the bottom table displays those with 2.0% gelatin content.


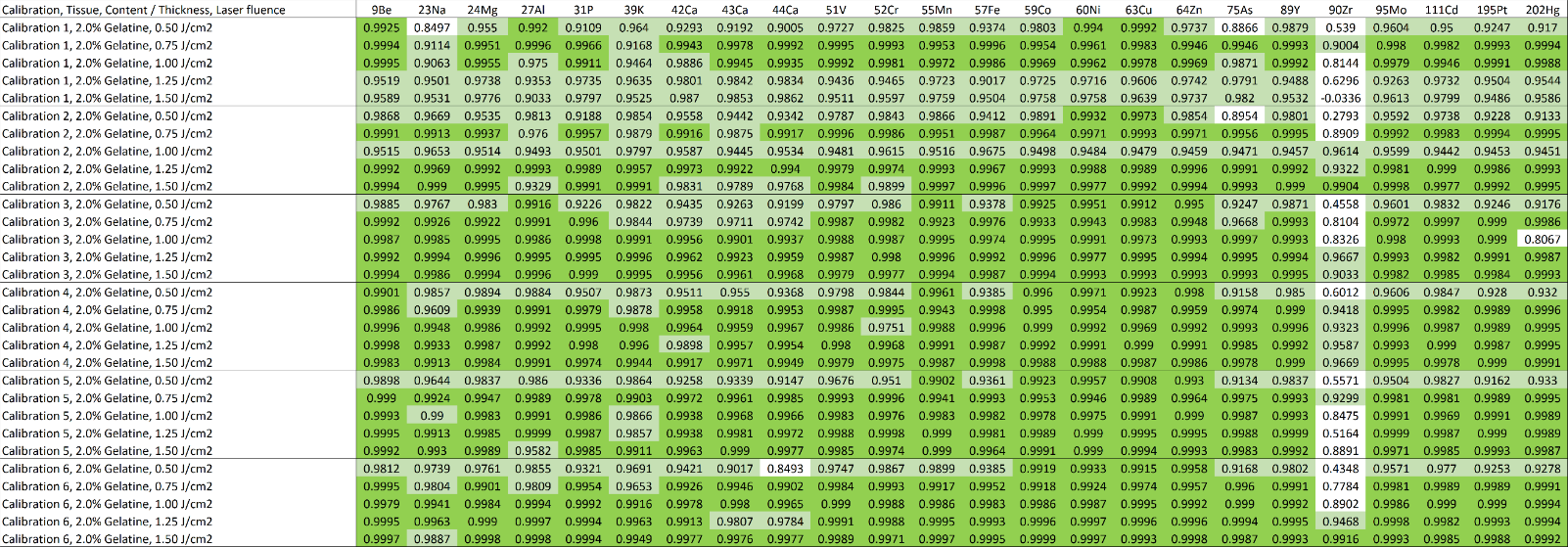


Table of the correlation coefficients for gelatin calibrations (5.0%, 7.0%, 8.0%)


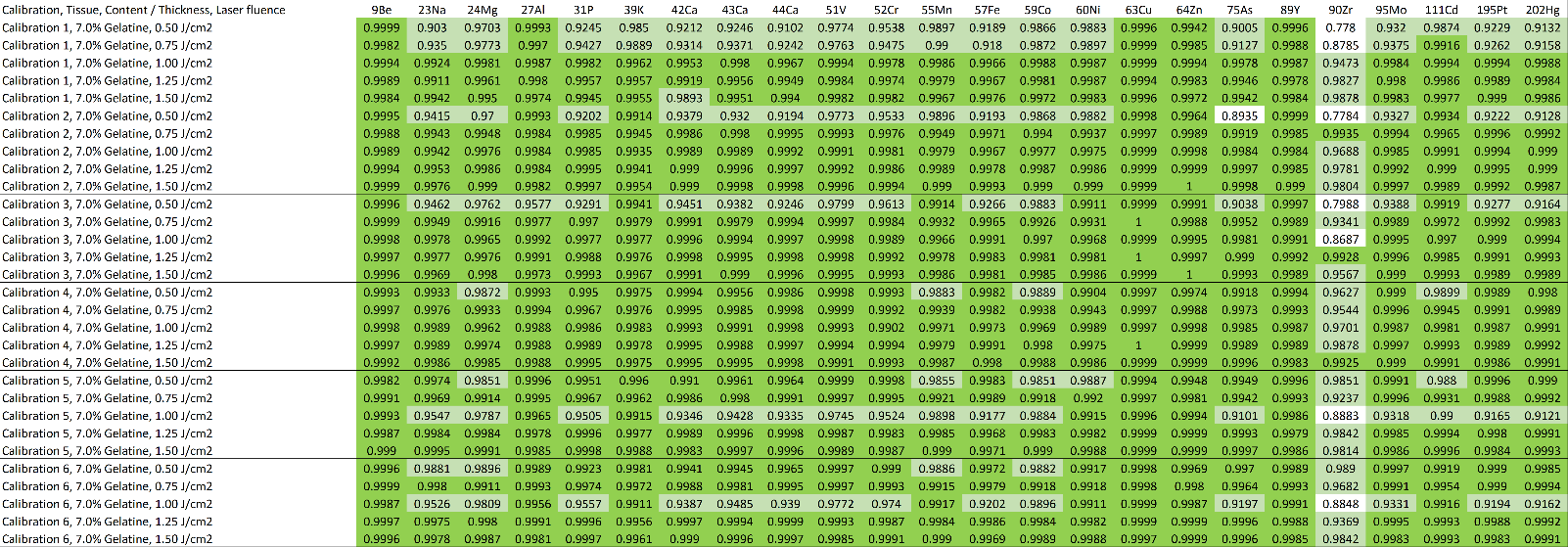

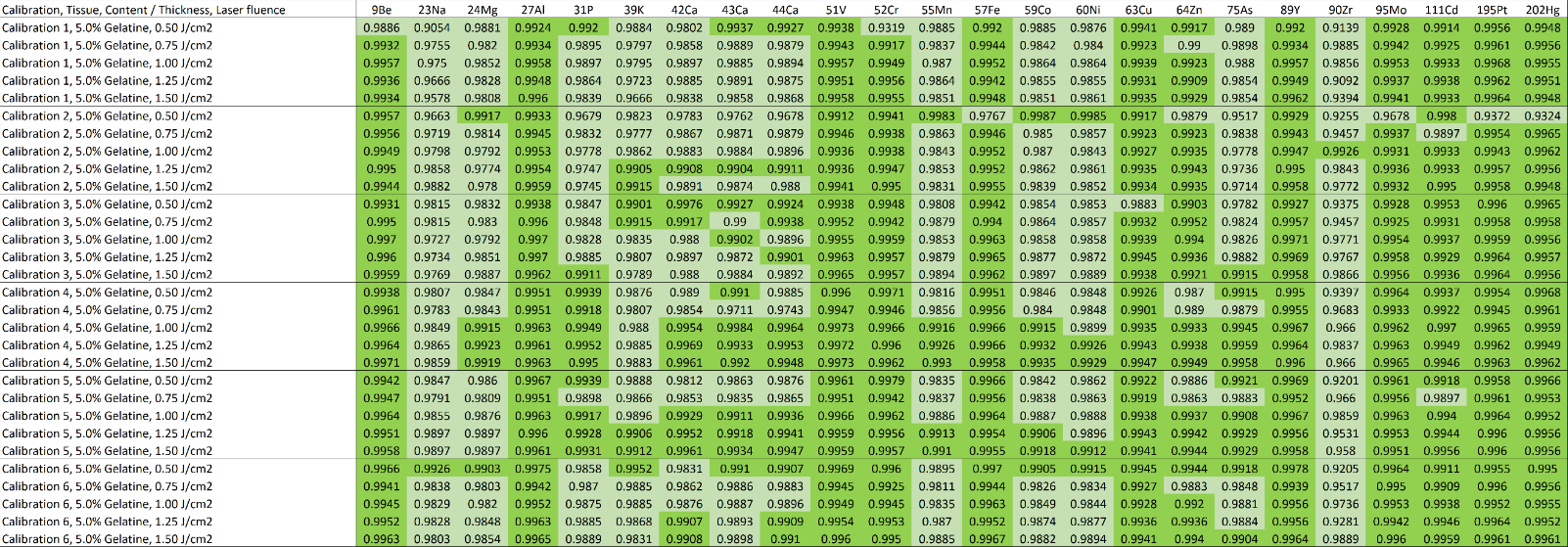


**Table 3b.** Depicted are six calibrations each with ablation energies of 0.50, 0.75, 1.00, 1.25 and 1.50 J/cm2. Light green indicates a correlation coefficient equal to or better than 0.9, while darker green signifies a correlation coefficient equal to or better than 0.99. The upper table displays the correlation coefficients of gelatin concentrations with 5.0% gelatin content, the middle table shows the correlation coefficients of gelatin concentrations with 7.0% gelatin content and the lower table shows the correlation coefficients of gelatine concentrations with 8.0% gelatine content.


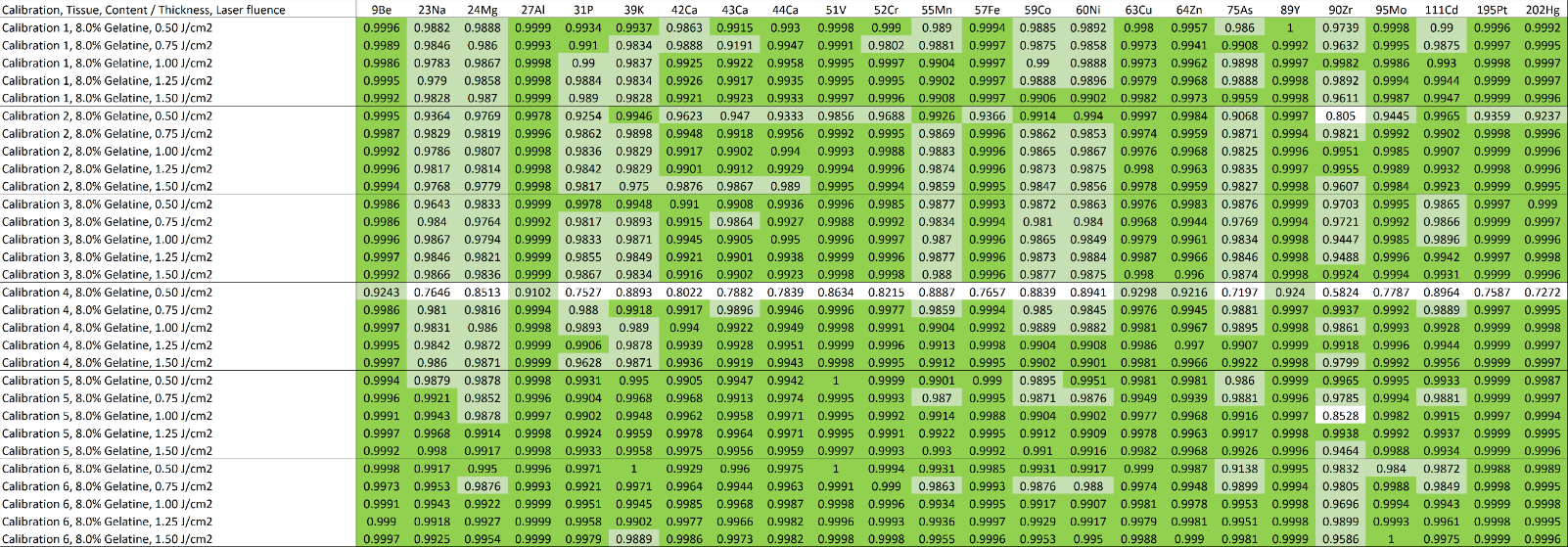


Table of the correlation coefficients for gelatin calibrations (9.0%) and calibrations with tissue homogenates (lung, liver)


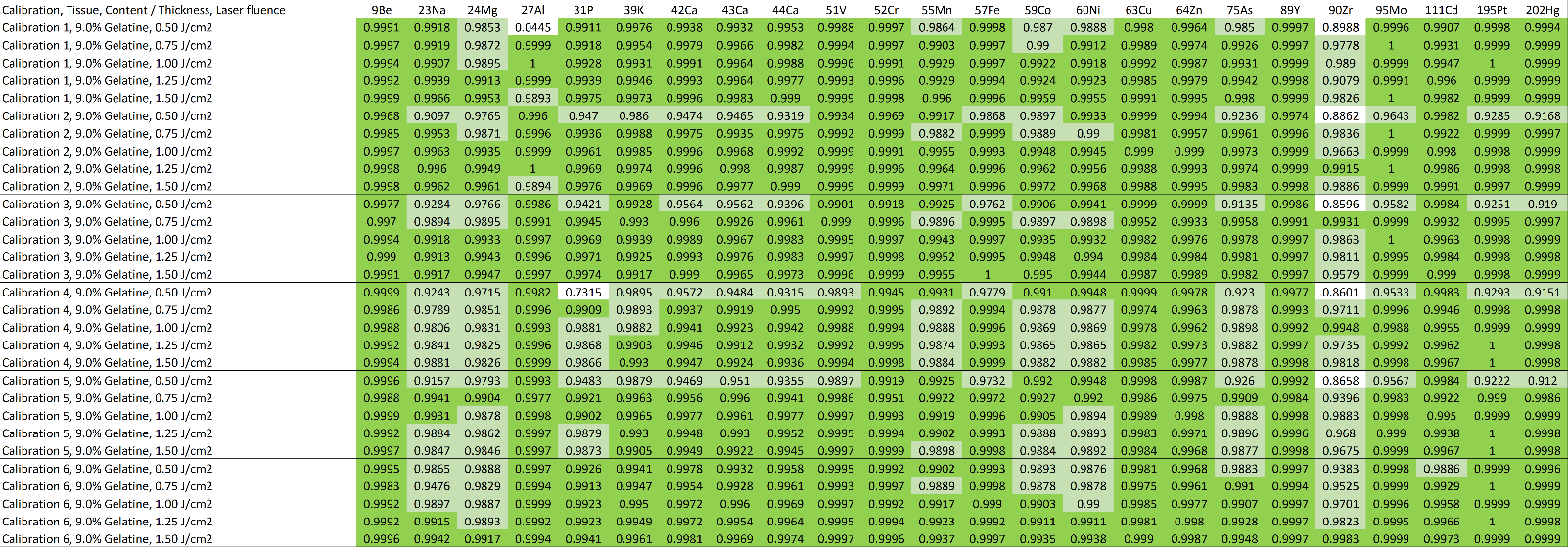


**Table 3c.** The upper table displays the correlation coefficients of six calibrations each with ablation energies of 0.50, 0.75, 1.00, 1.25 and 1.50 J/cm2, using gelatin with a 9.0% content. The middle table shows the correlation coefficients of calibrations using lung tissue homogenates at section thicknesses of 10, 20 and 30 µm. Two calibrations were conducted for each section thickness based on the energy settings mentioned above. The table below provides the same information with liver tissue homogenates. Light green indicates a correlation coefficient equal to or greater than 0.9, while darker green signifies a correlation coefficient better than or equal to 0.99. The lower table displays the correlation coefficients of gelatine concentrations with an 8.0% gelatin content.


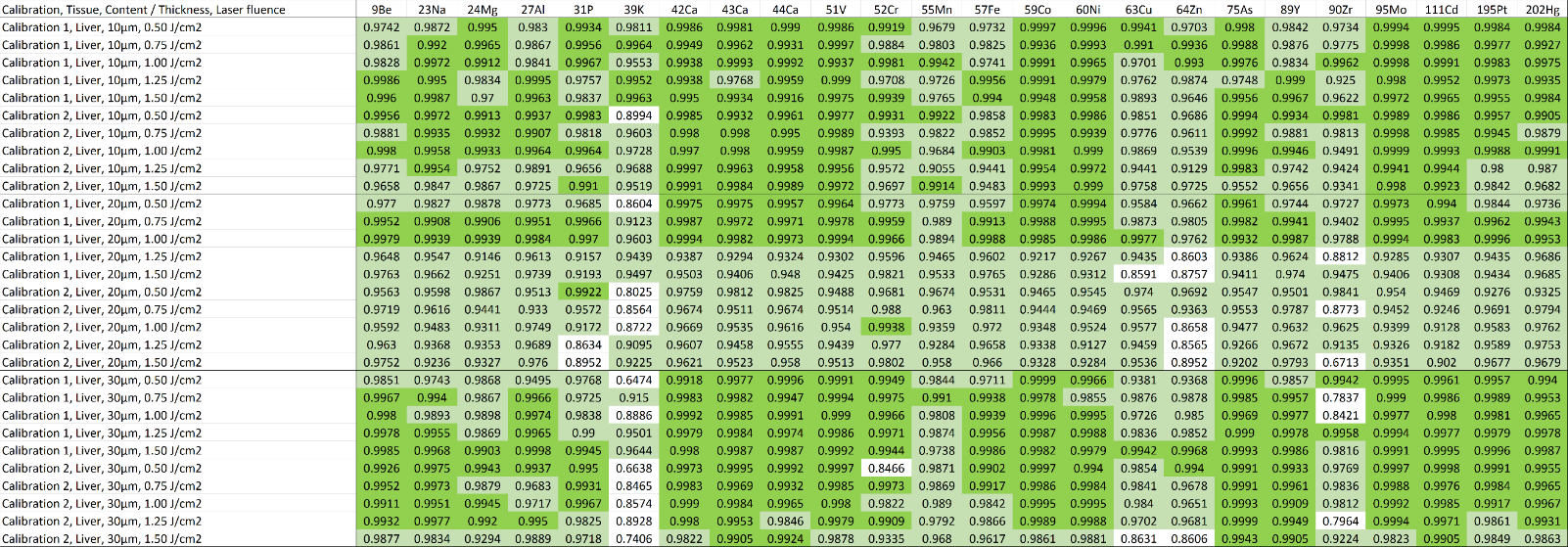

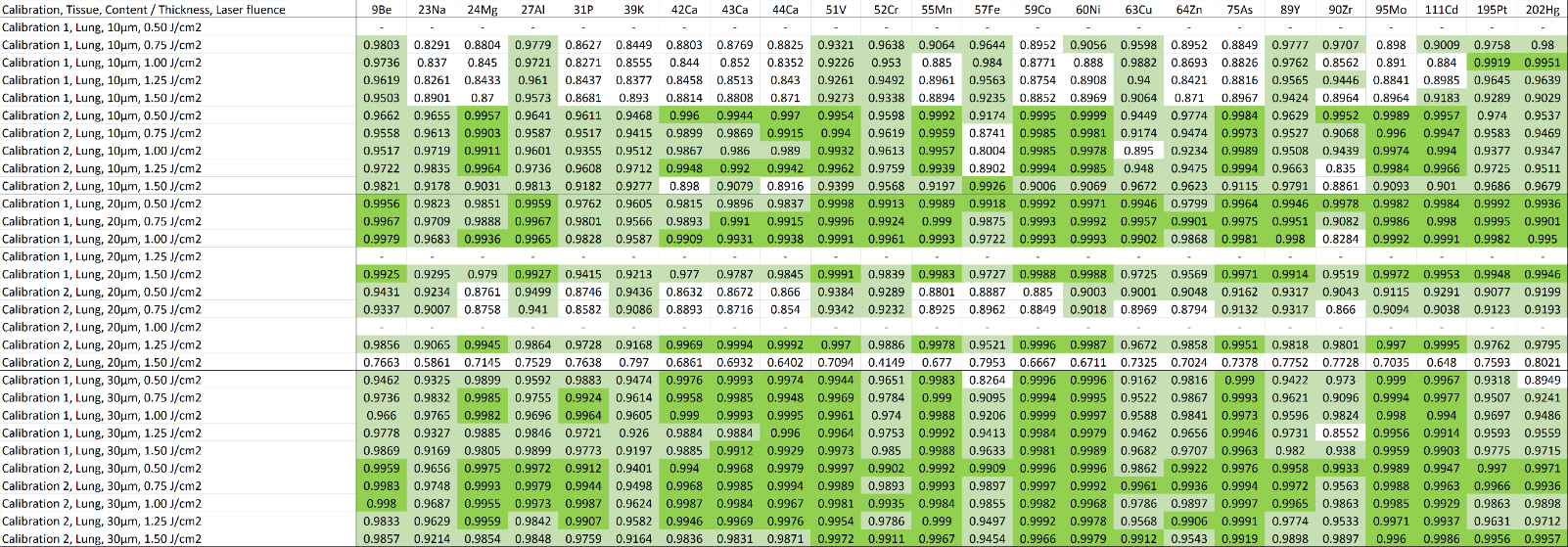


Evaluations (median/without de-spiking) of all measured isotopes in lung homogenate samples via gelatin calibrations

**Fig 2a.** The figure shows box-whisker plots of the median values of all measured isotopes in the lung homogenate samples without de-spiking (element spikes), evaluated with gelatin calibrations (n = 5,400). The upper illustration displays isotopes of the lower concentration range (section 2.2.1). The lower left image shows all isotopes of the 10-fold higher concentration range (27Al, 64Zn). The middle illustration below displays isotopes of the 100-fold higher concentration range (24Mg, 42Ca, 44Ca, 57Fe) and the lower right illustration shows isotopes of the 1,000-fold higher concentration range, which includes 23Na, 31P, and 39K.

C: Calibration, S: Sample


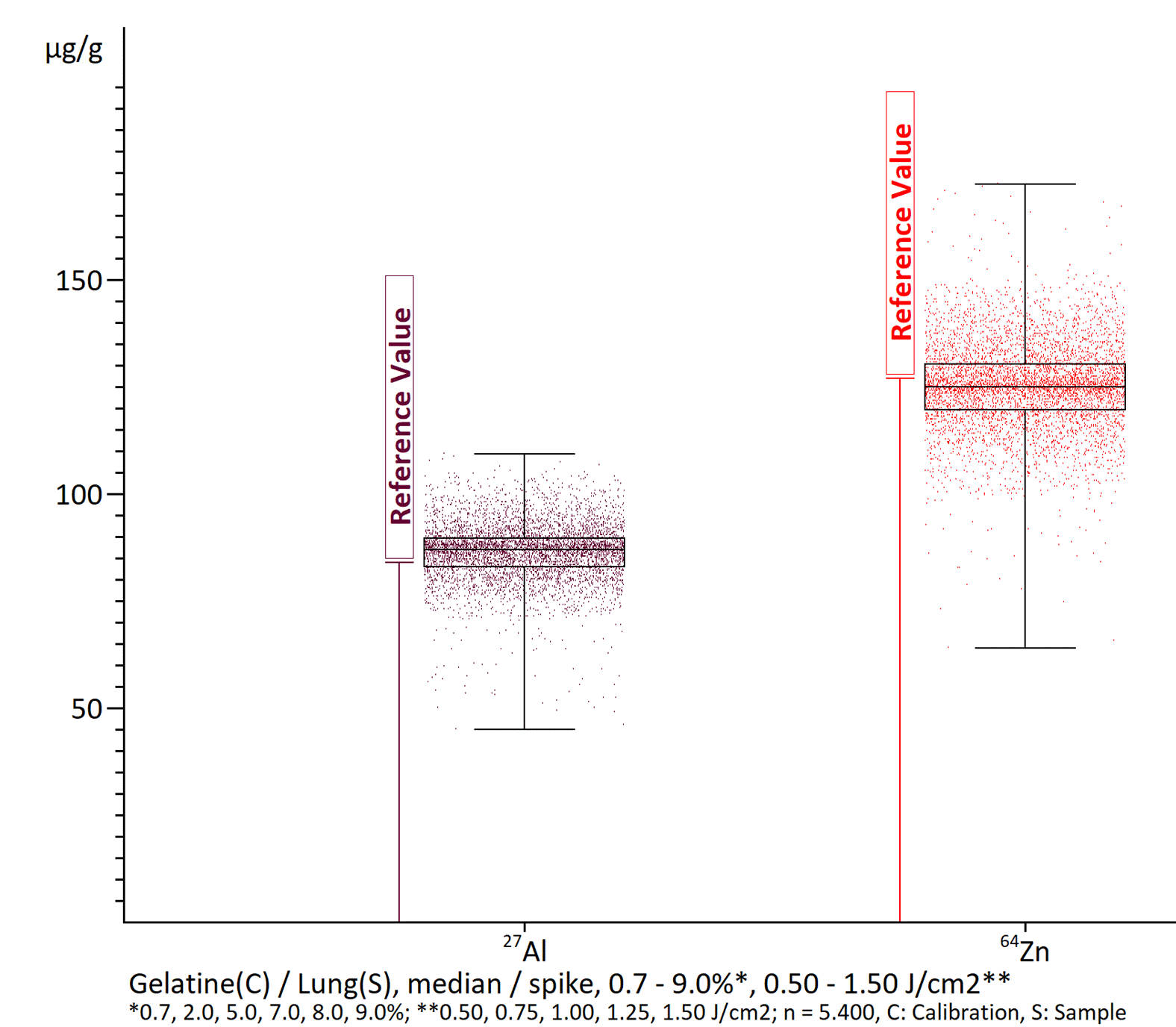

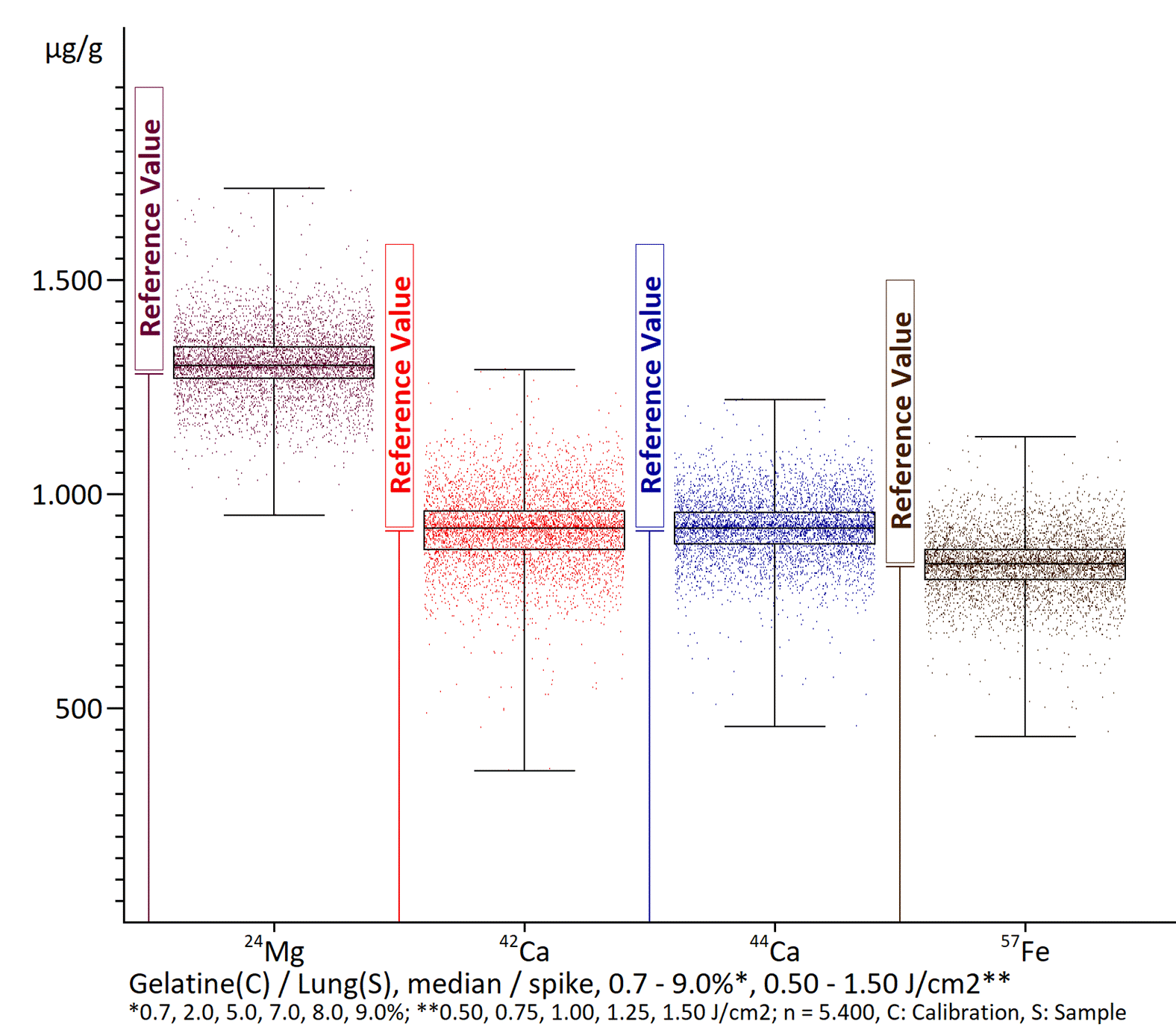

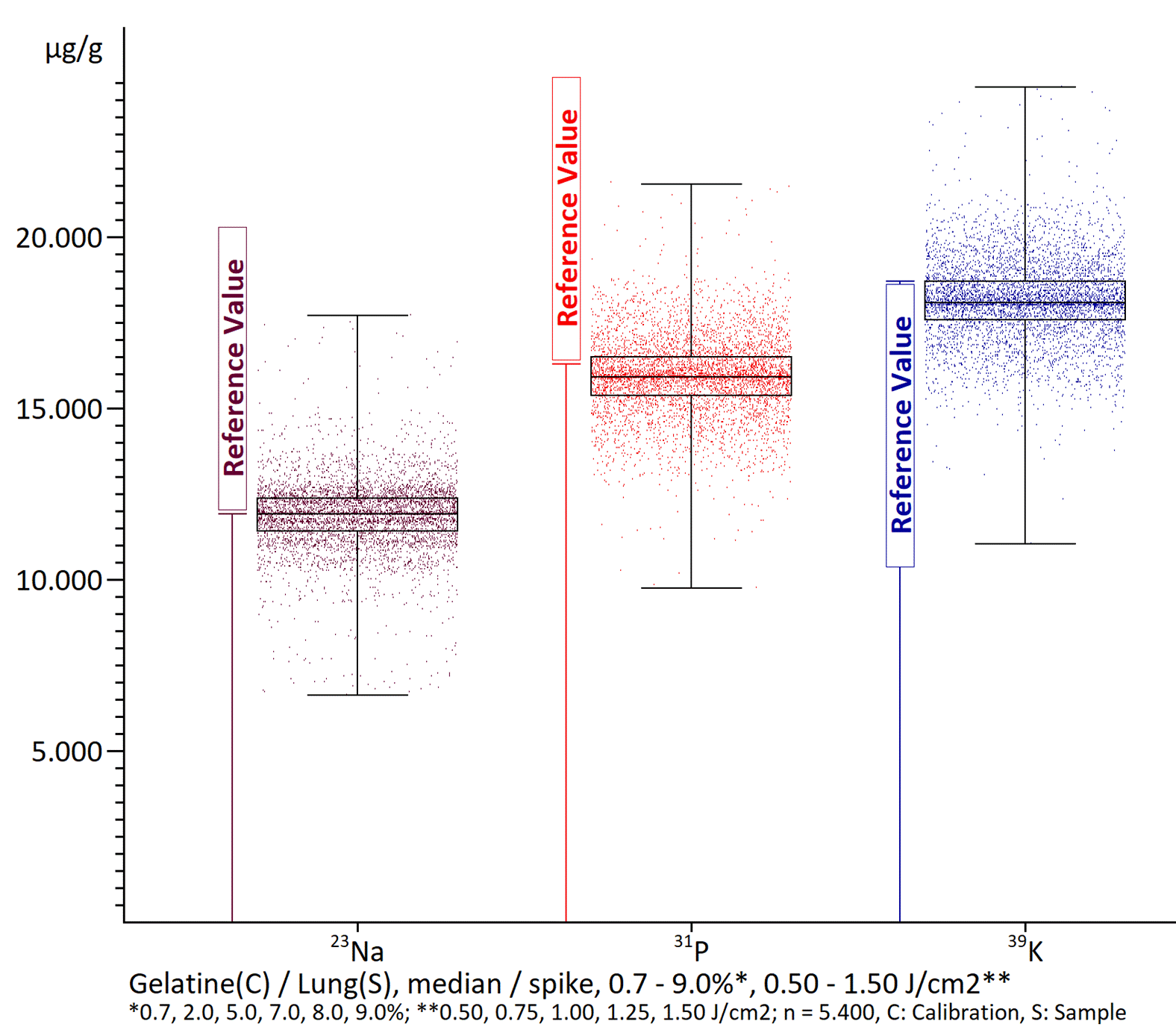

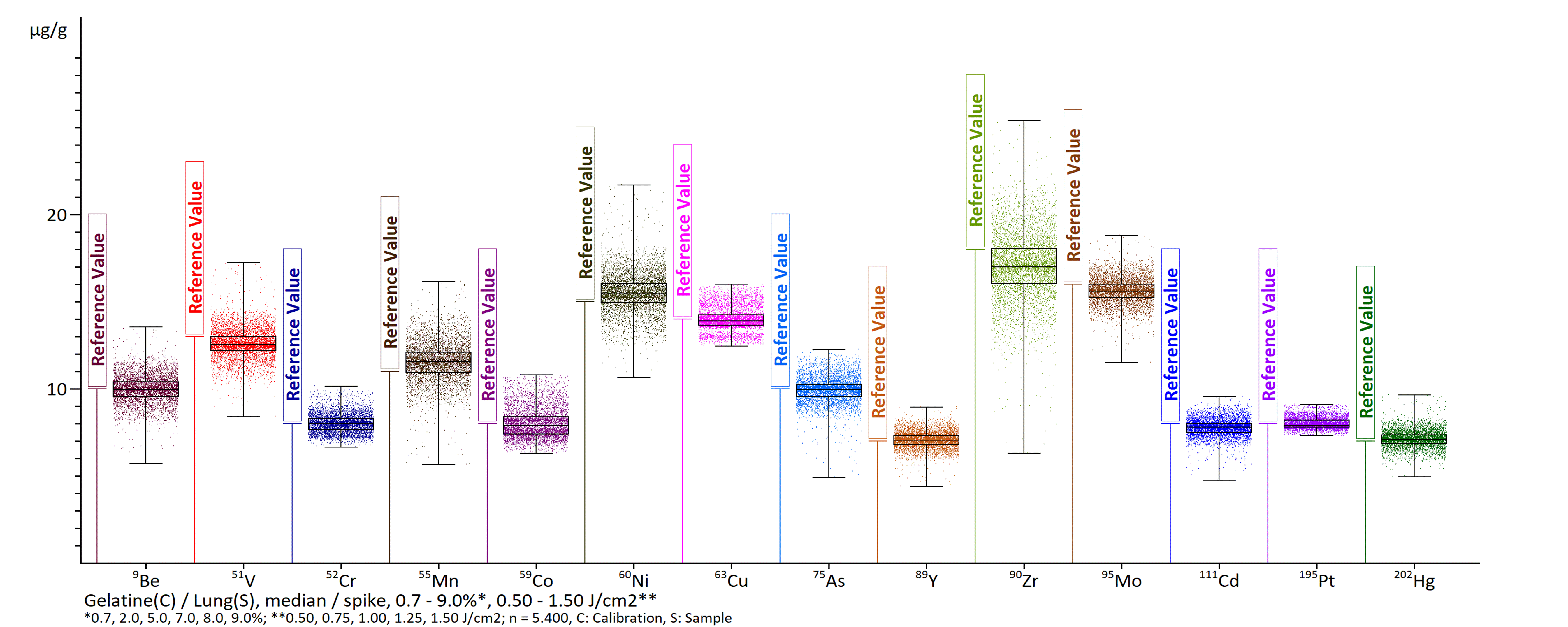


Evaluations (median/without de-spiking) of all measured isotopes in lung homogenate samples via lung homogenate calibrations

**Fig 2b.** The figure shows box-whisker plots of the median values of all measured isotopes in lung homogenate samples without de-spiking (element spikes) that were assessed using lung homogenate calibrations (n = 900). The top illustration represents isotopes within the lower concentration range (section 2.2.1). The lower left image shows isotopes in a range that is 10 times higher in concentration (27Al, 64Zn). The middle illustration below displays isotopes in a range that is 100 times higher in concentration (24Mg, 42Ca, 44Ca, 57Fe), while the lower right illustration shows isotopes in a range that is 1,000 times higher in concentration, including 23Na, 31P, and 39K.

C: Calibration, S: Sample


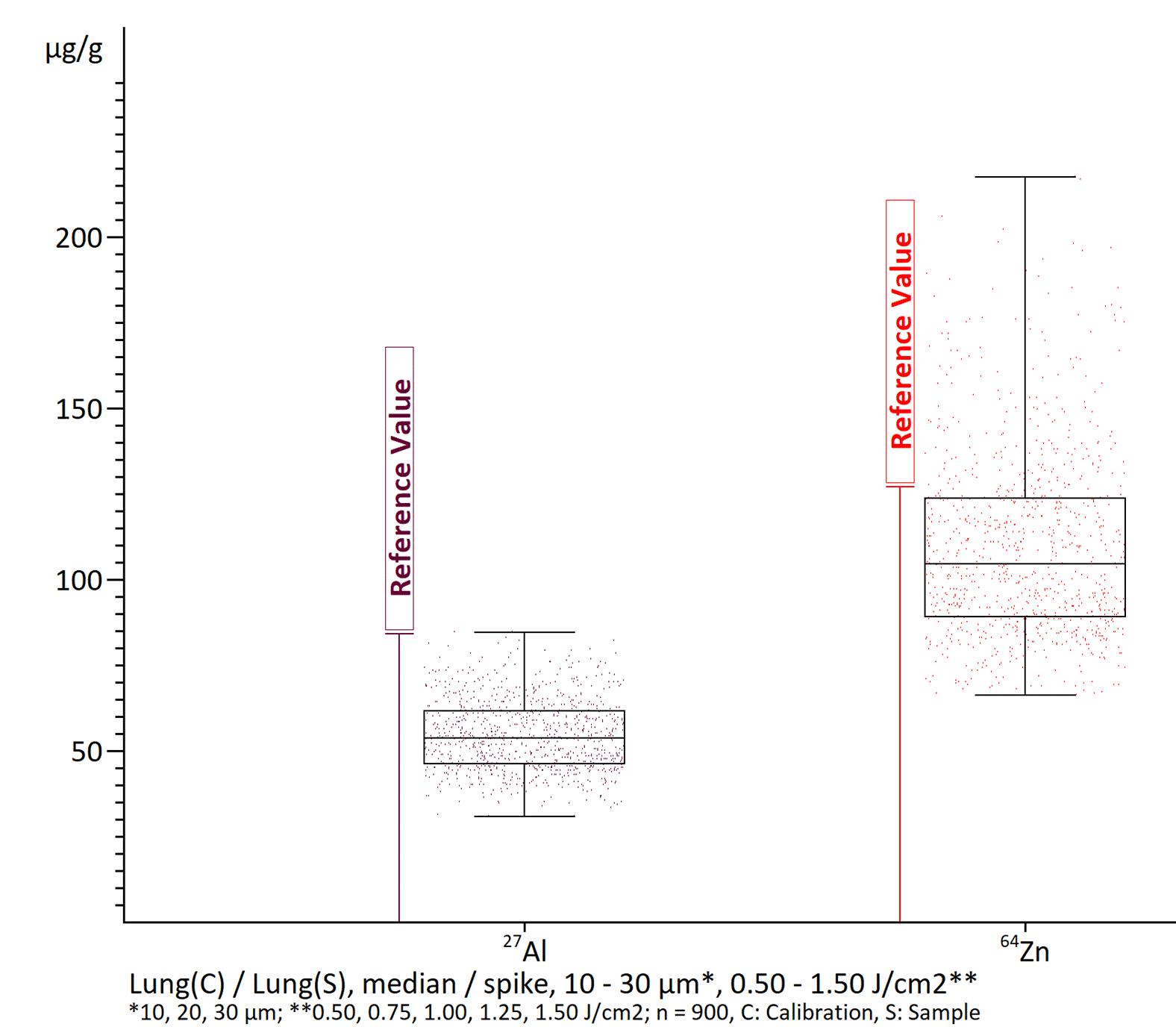

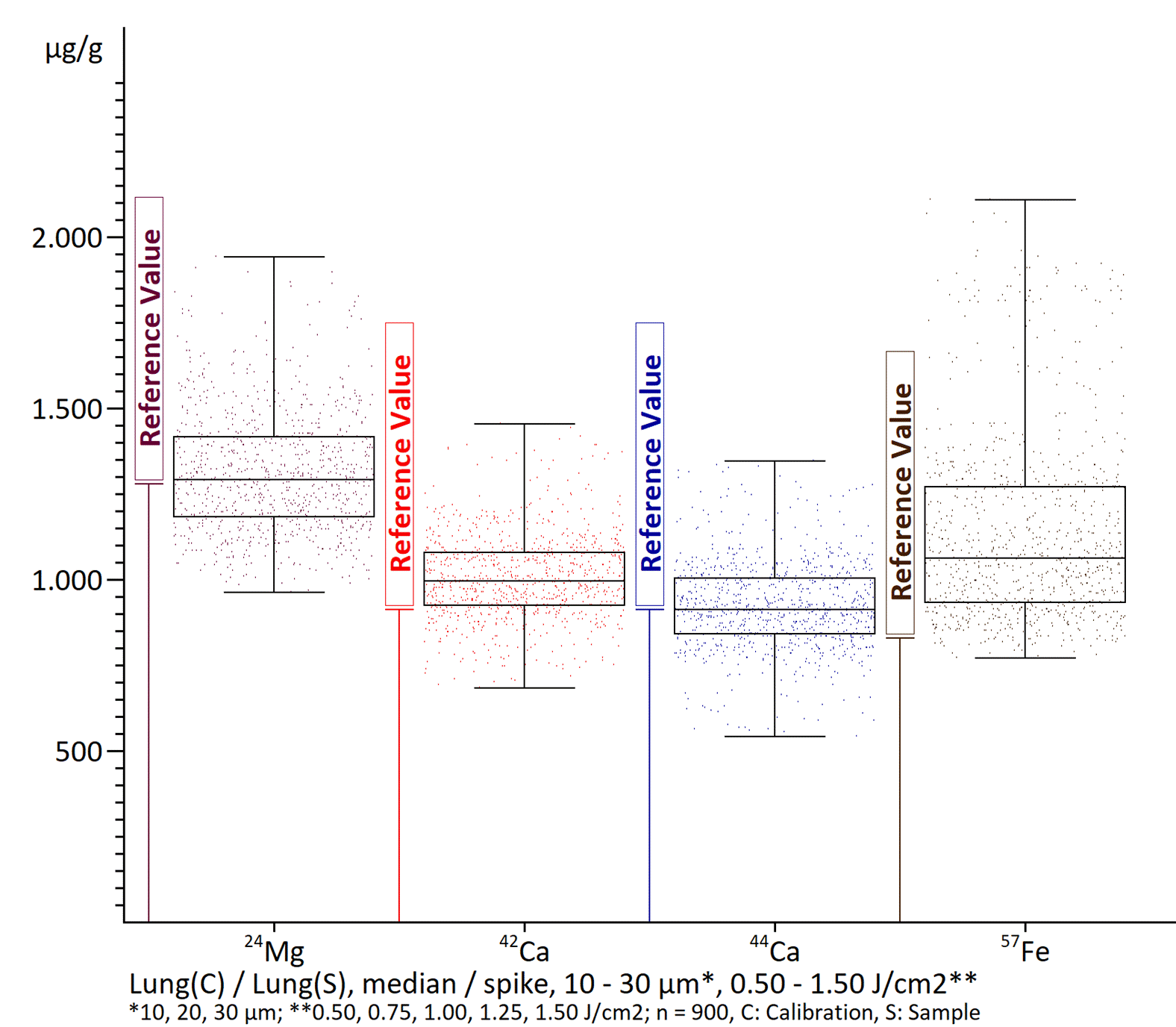

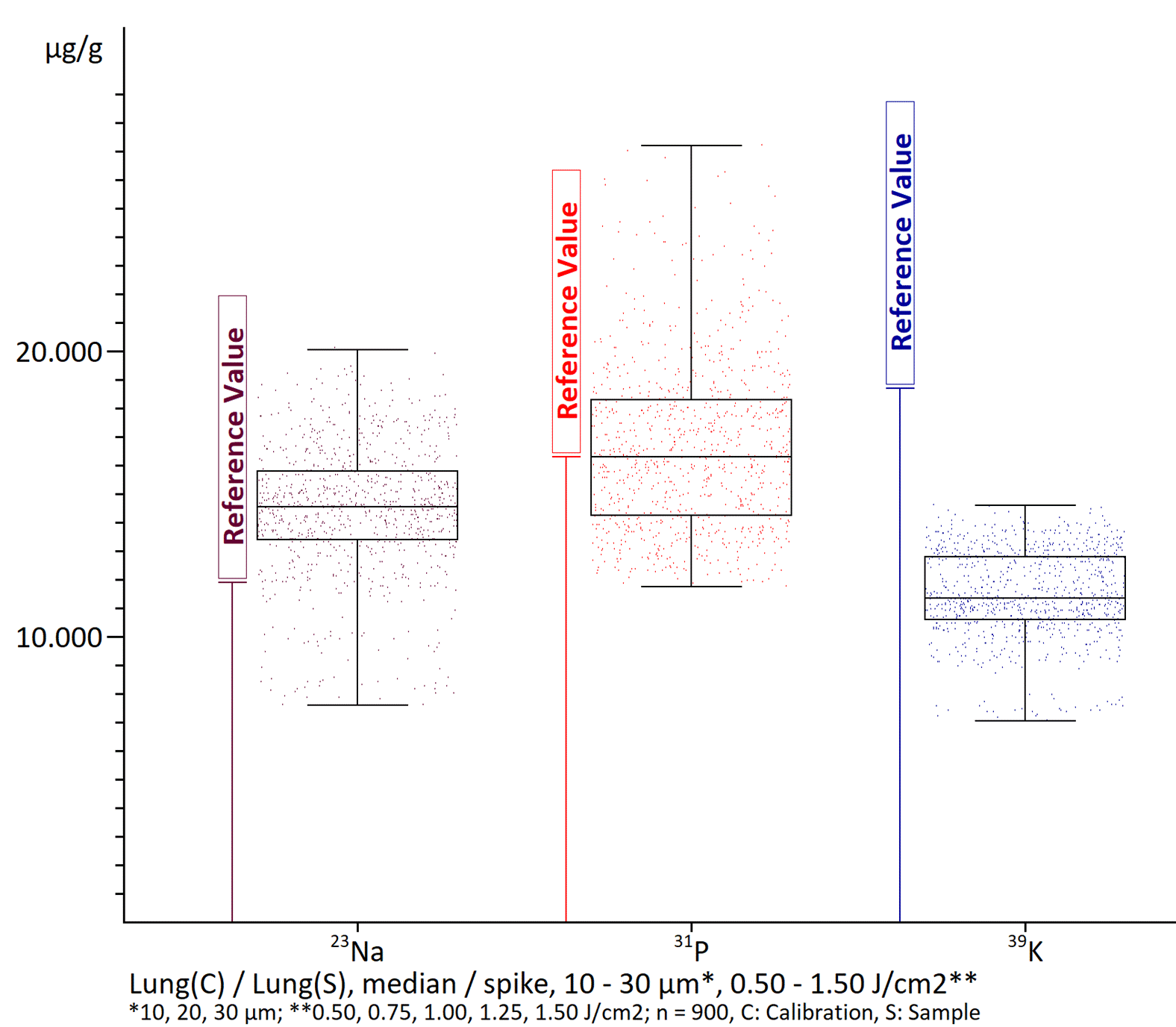

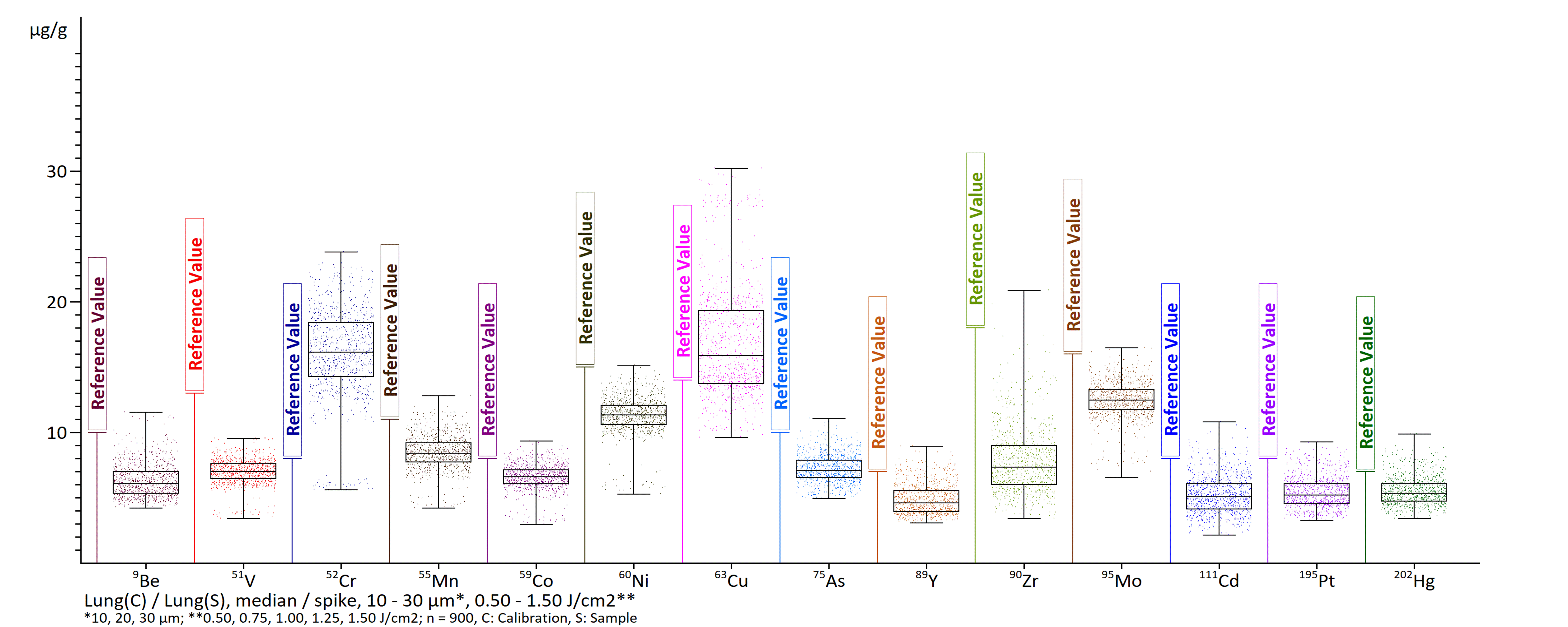


Evaluations (median/without de-spiking) of all measured isotopes in lung homogenate samples via liver homogenate calibrations

**Fig 2c.** The figure shows box-whisker plots of the median values of all measured isotopes in the lung homogenate samples without de-spiking (element spikes), evaluated with liver homogenate calibrations (n = 900). The upper illustration displays isotopes of the lower concentration range (section 2.2.1). The lower left image shows all isotopes of the 10-fold higher concentration range (27Al, 64Zn). The middle illustration below displays isotopes of the 100-fold higher concentration range (24Mg, 42Ca, 44Ca, 57Fe) and the lower right illustration shows isotopes of the 1,000-fold higher concentration range, which includes 23Na, 31P, and 39K.

C: Calibration, S: Sample


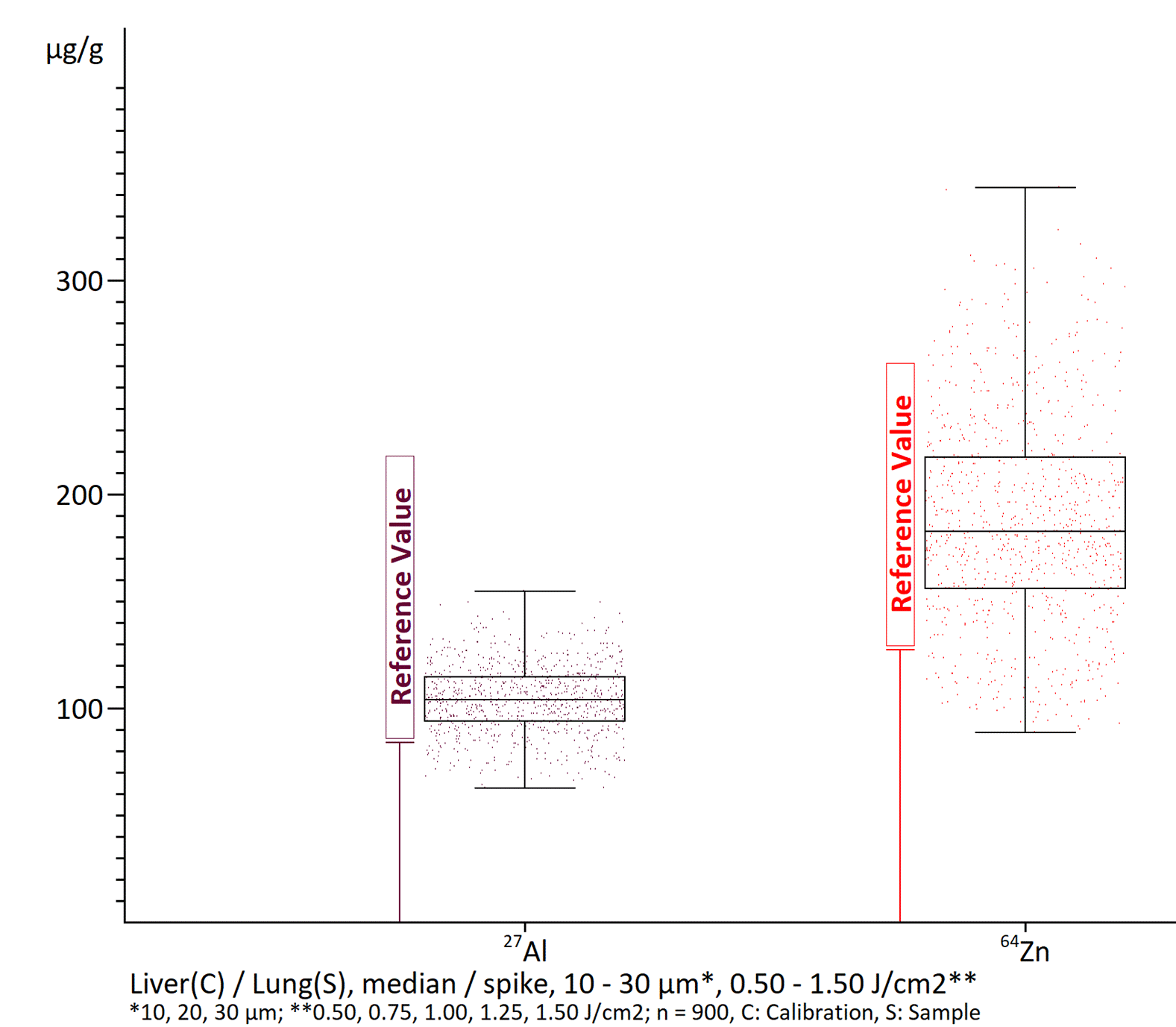

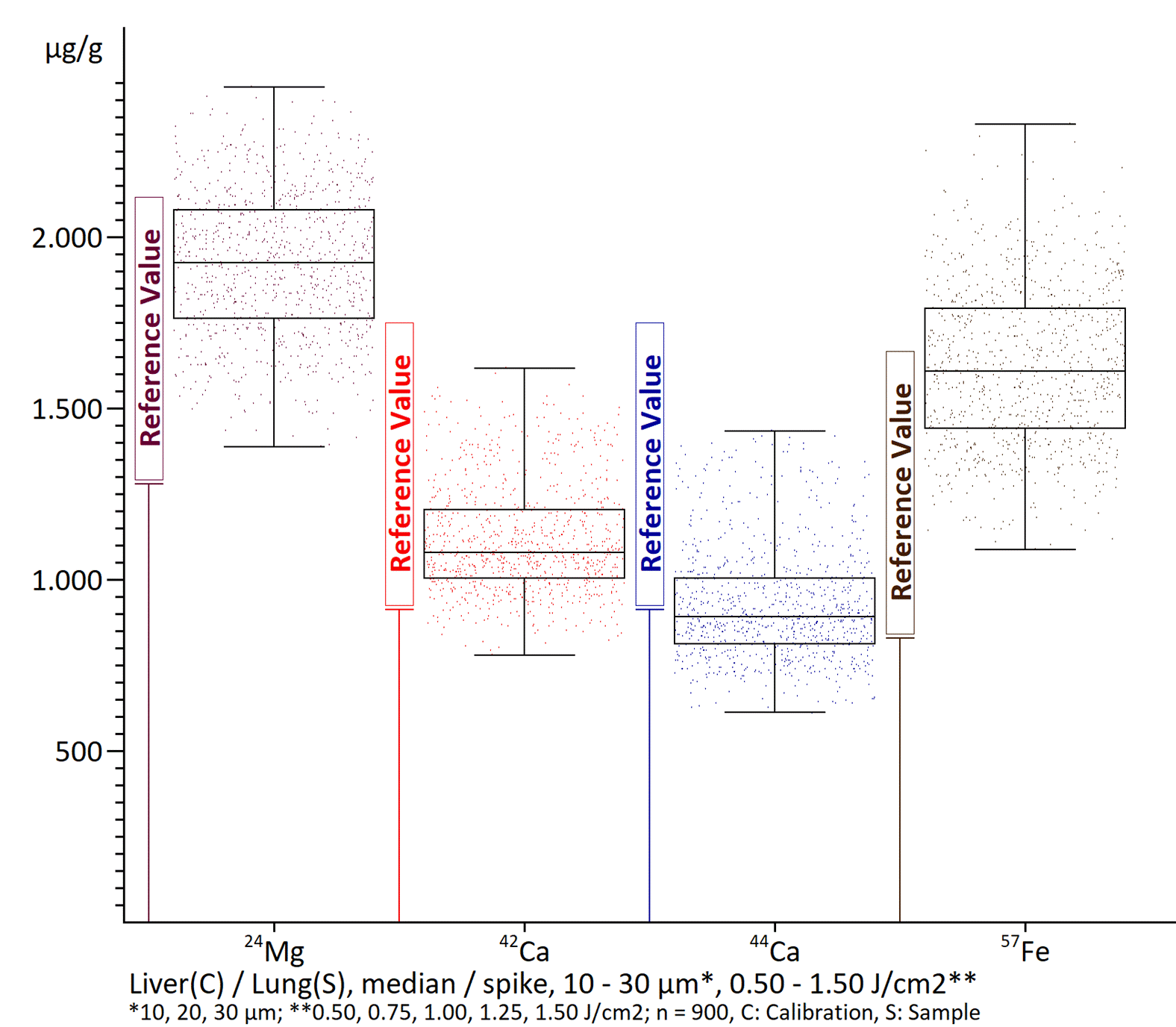

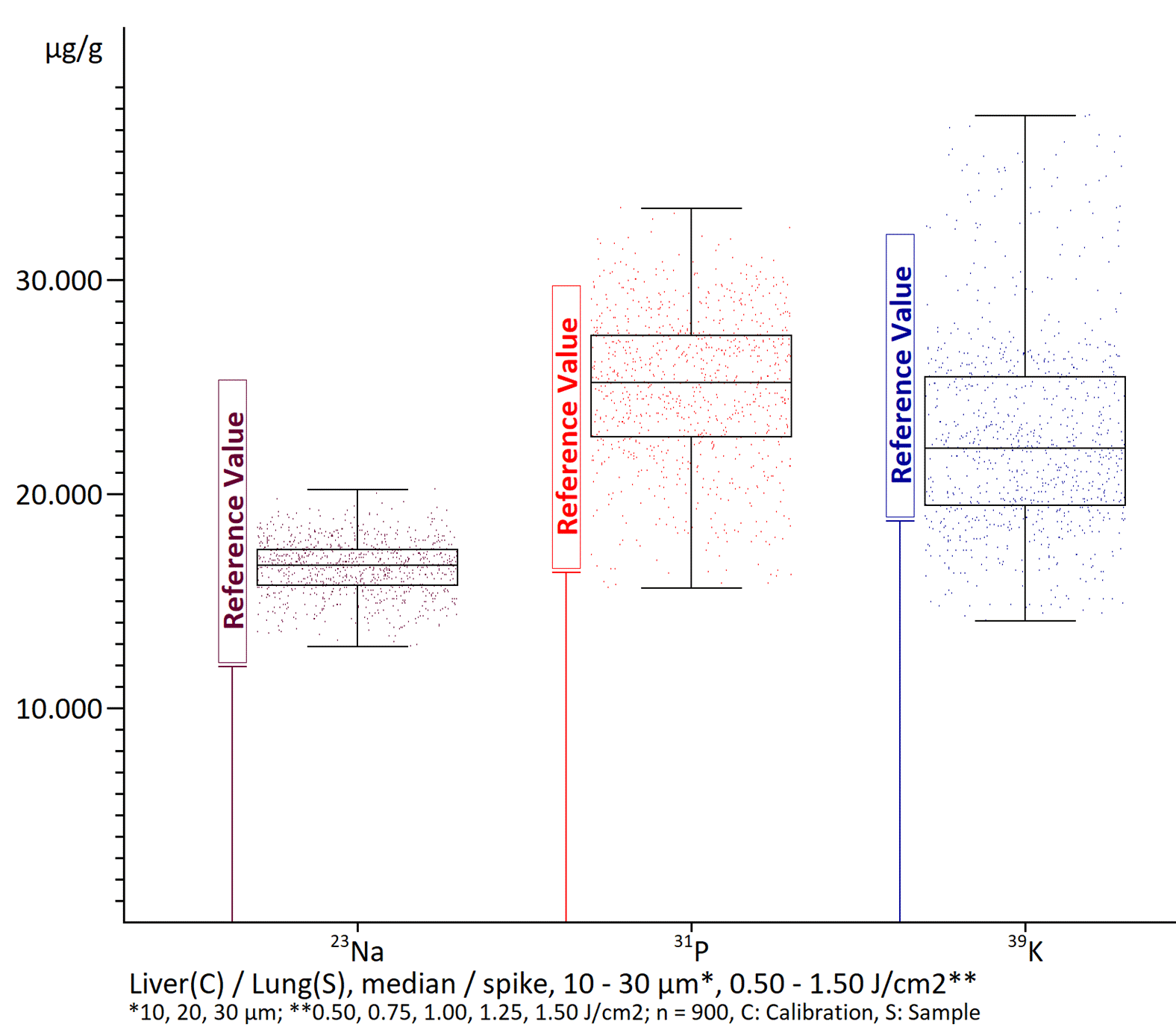

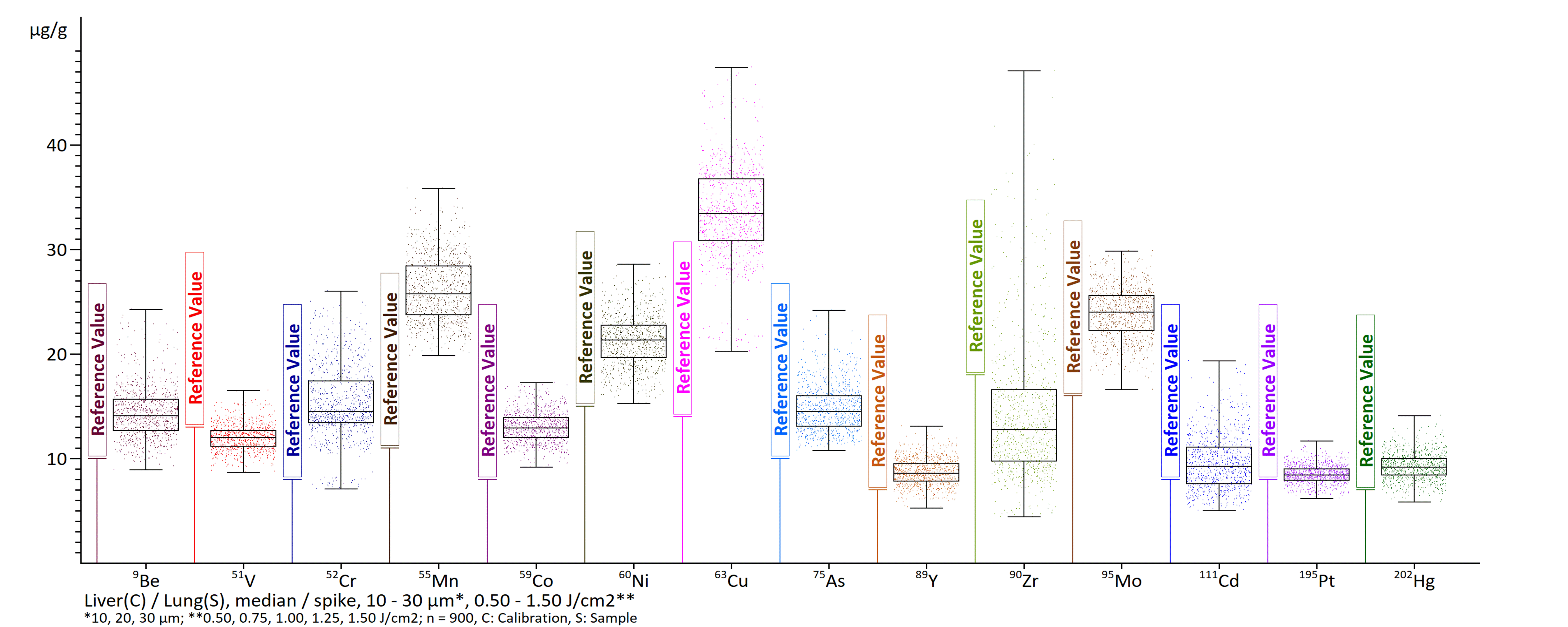


Evaluations (median/without de-spiking) of all measured isotopes in liver homogenate samples via gelatin calibrations

**Fig 3a.** The figure shows box-whisker plots of the median values of all measured isotopes in liver homogenate samples without de-spiking (element spikes) that were assessed using gelatin calibrations (n = 5.400). The top illustration represents isotopes within the lower concentration range (section 2.2.1). The lower left image shows isotopes in a range that is 10 times higher in concentration (27Al, 64Zn). The middle illustration below displays isotopes in a range that is 100 times higher in concentration (24Mg, 42Ca, 44Ca, 57Fe), while the lower right illustration shows isotopes in a range that is 1,000 times higher in concentration, including 23Na, 31P, and 39K.

C: Calibration, S: Sample


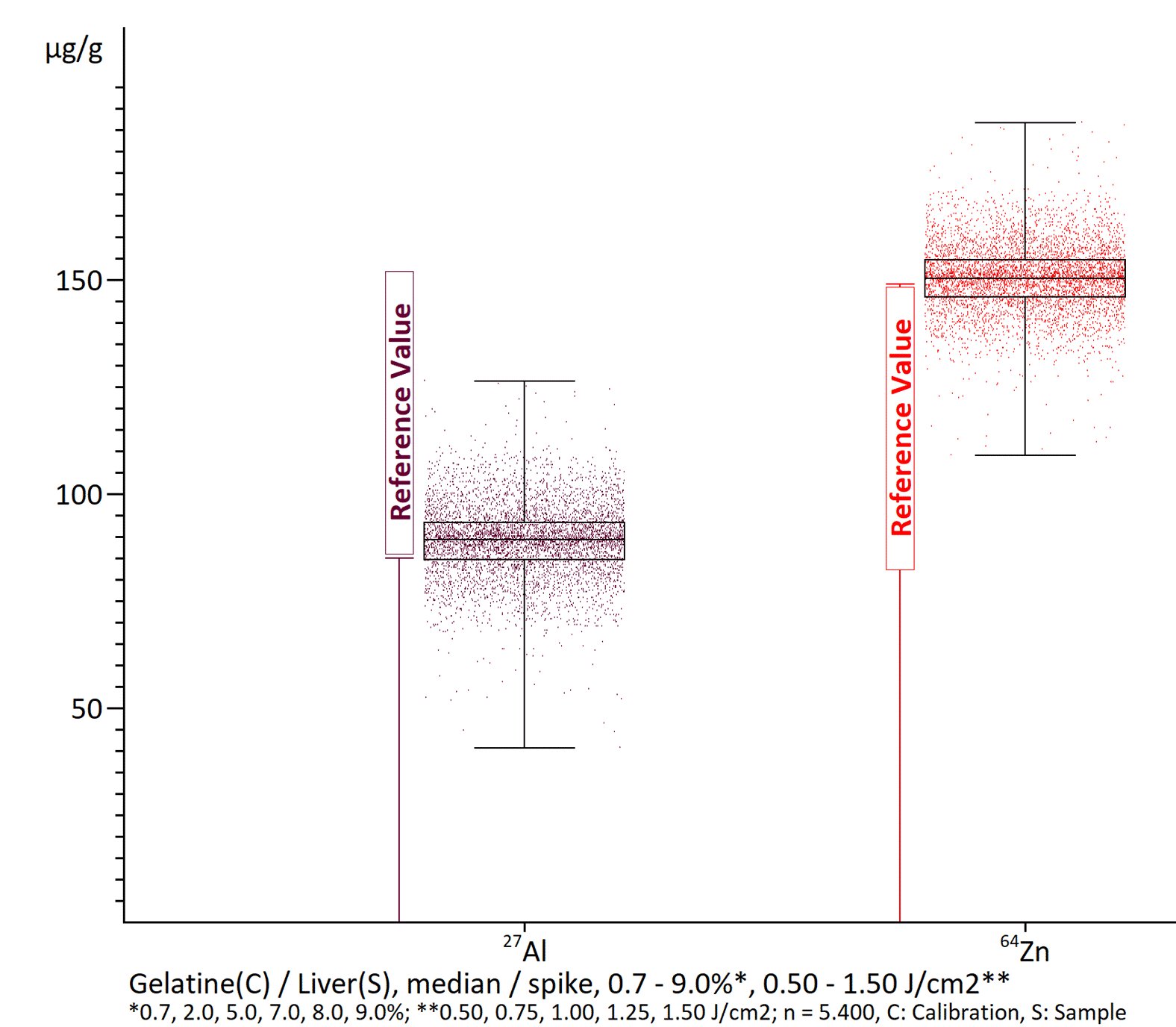

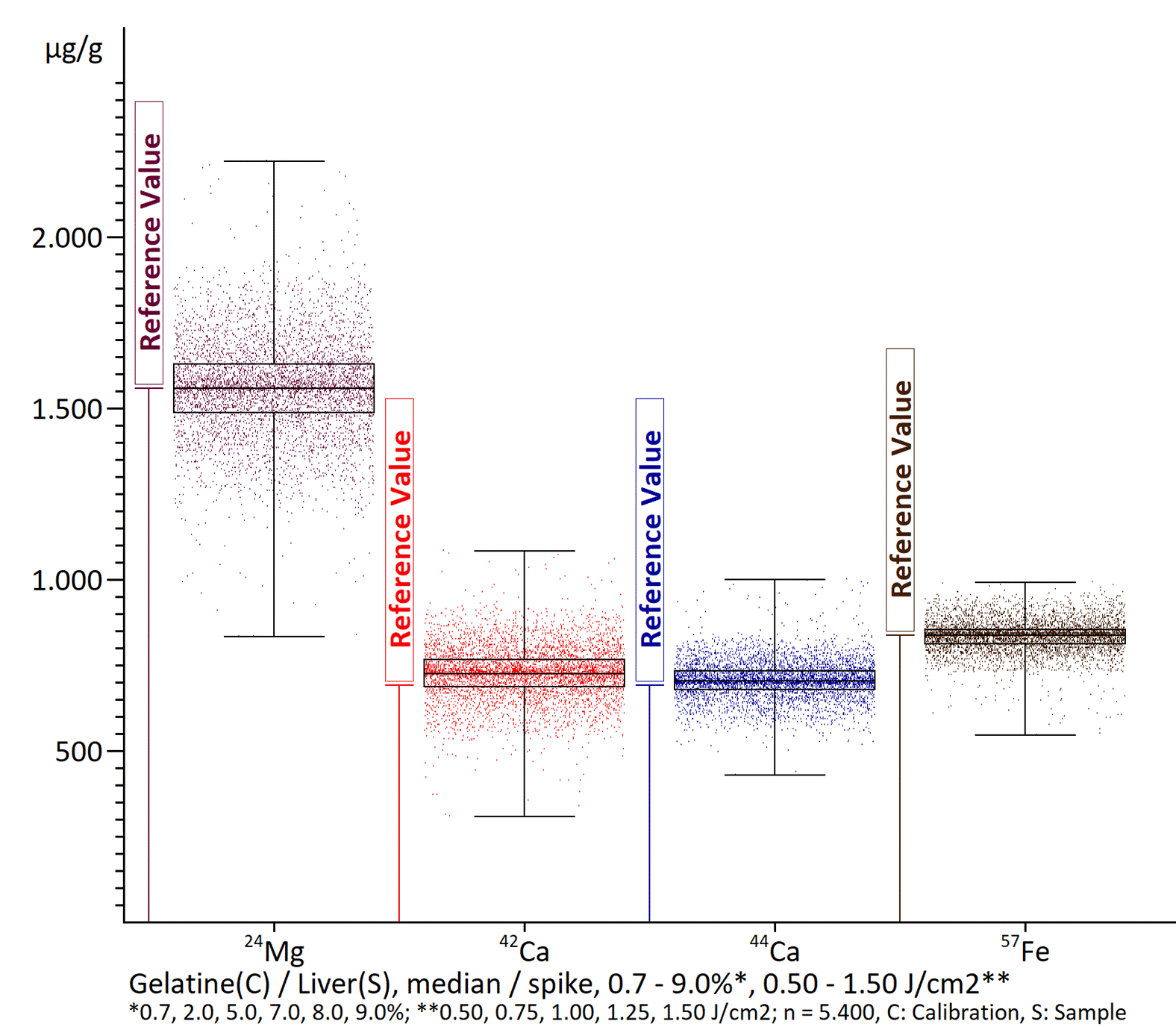

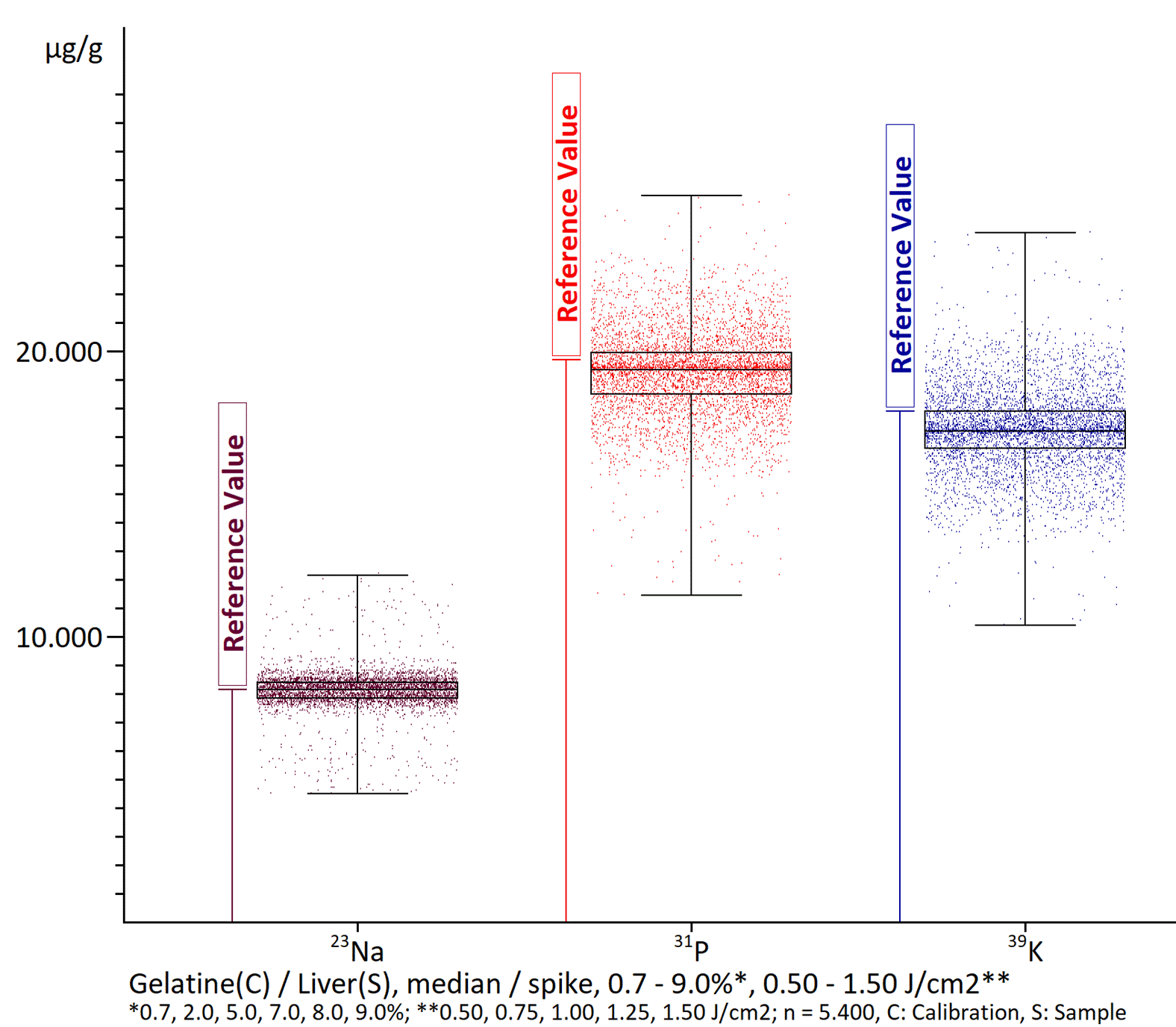

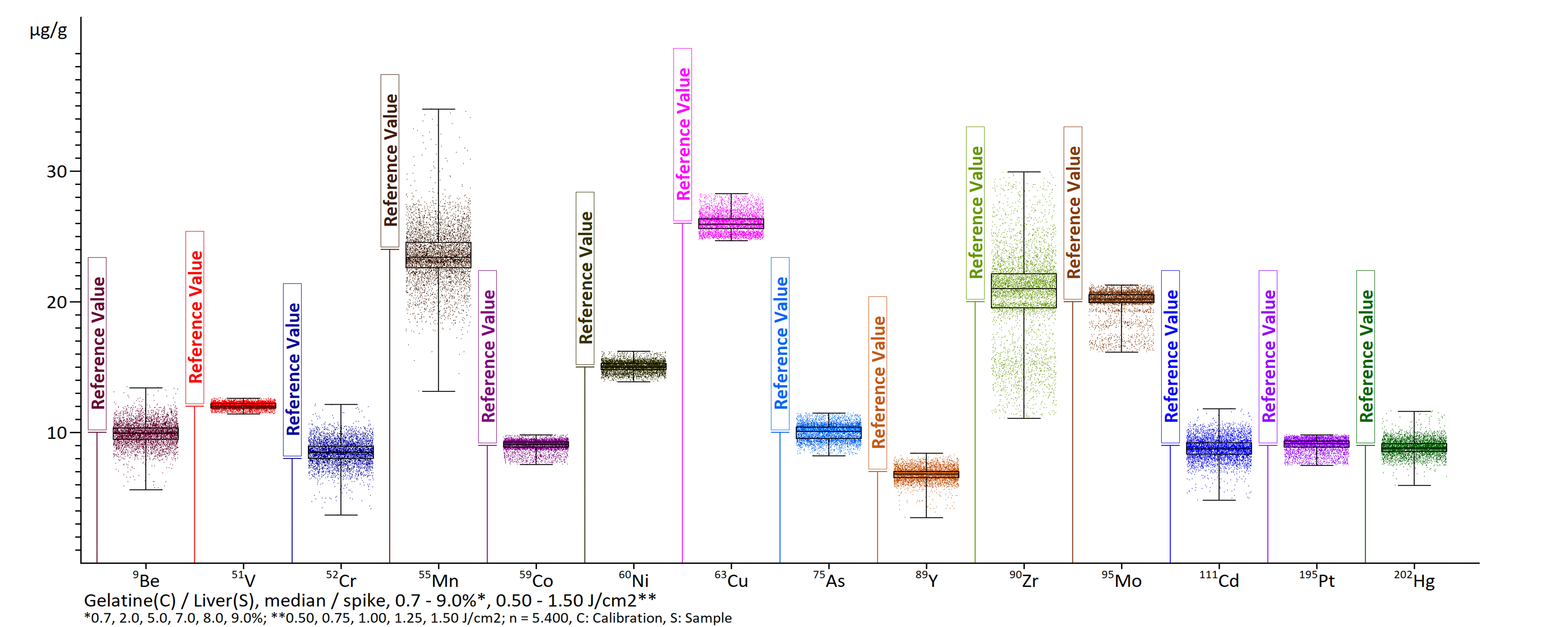


Evaluations (median/without de-spiking) of all measured isotopes in liver homogenate samples via lung homogenate calibrations

**Fig 3b.** The figure shows box-whisker plots of the median values of all measured isotopes in the liver homogenate samples without de-spiking (element spikes), evaluated with lung homogenate calibrations (n = 900). The upper illustration displays isotopes of the lower concentration range (section 2.2.1). The lower left image shows all isotopes of the 10-fold higher concentration range (27Al, 64Zn). The middle illustration below displays isotopes of the 100-fold higher concentration range (24Mg, 42Ca, 44Ca, 57Fe) and the lower right illustration shows isotopes of the 1,000-fold higher concentration range, which includes 23Na, 31P, and 39K.

C: Calibration, S: Sample


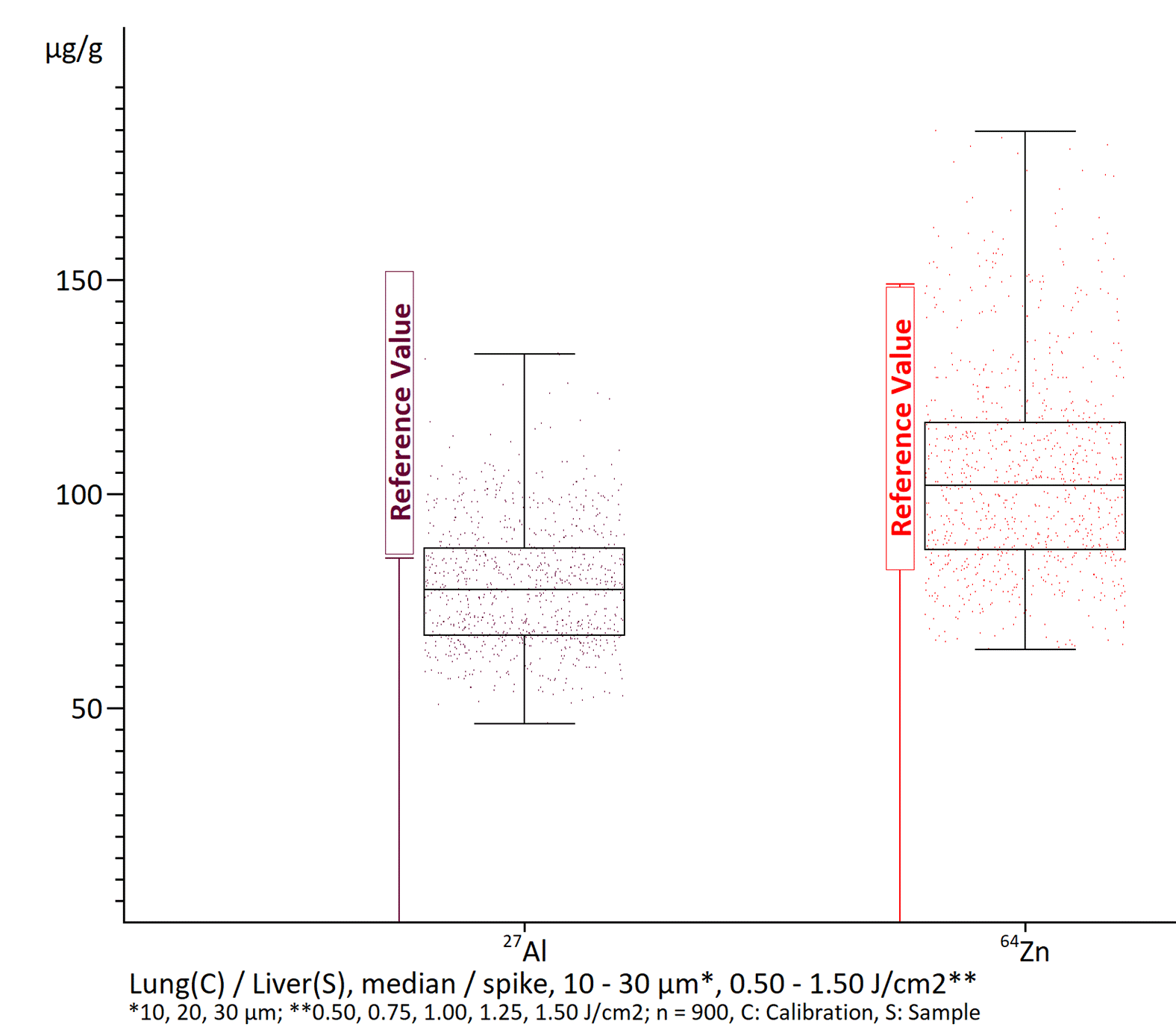

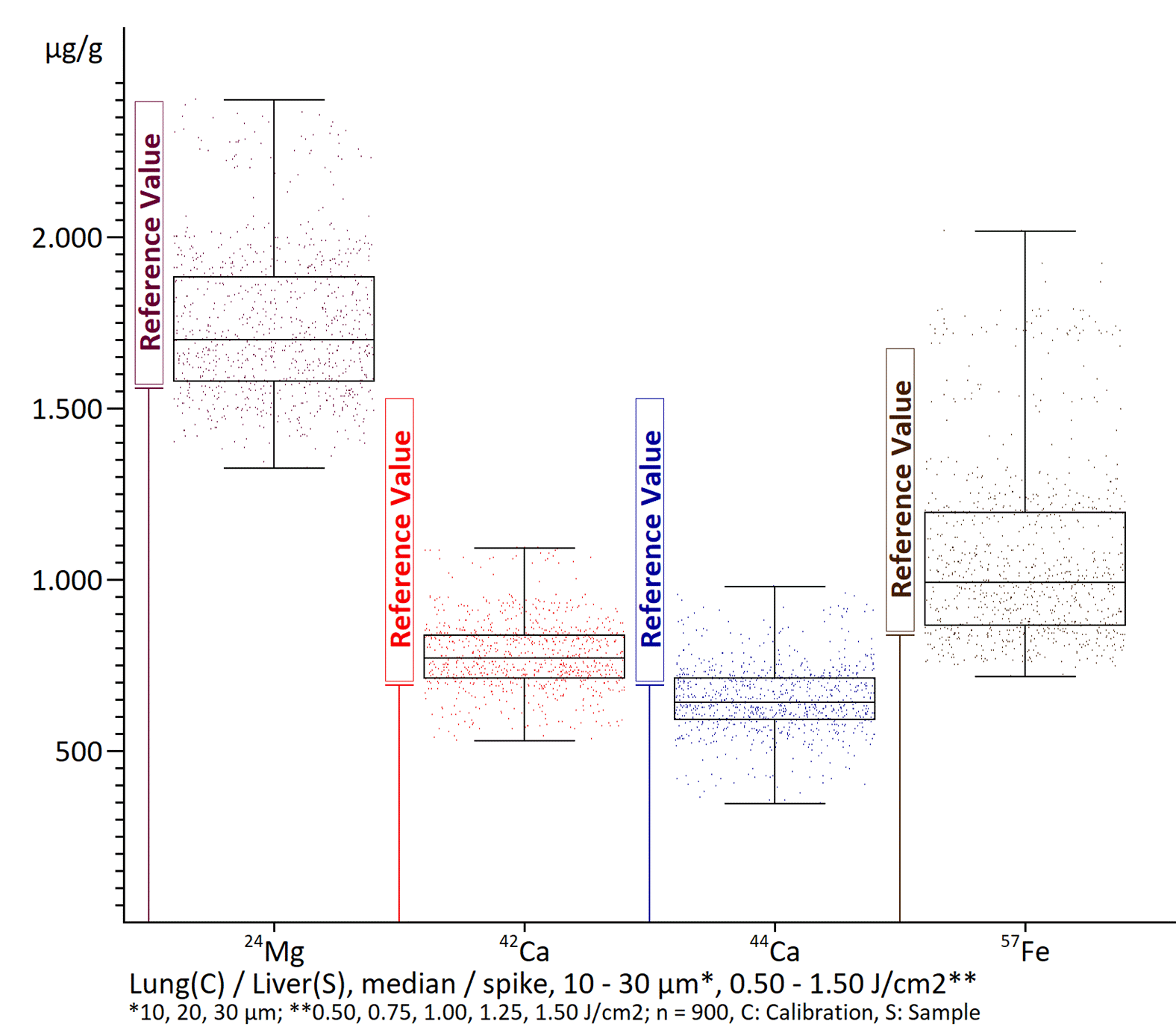

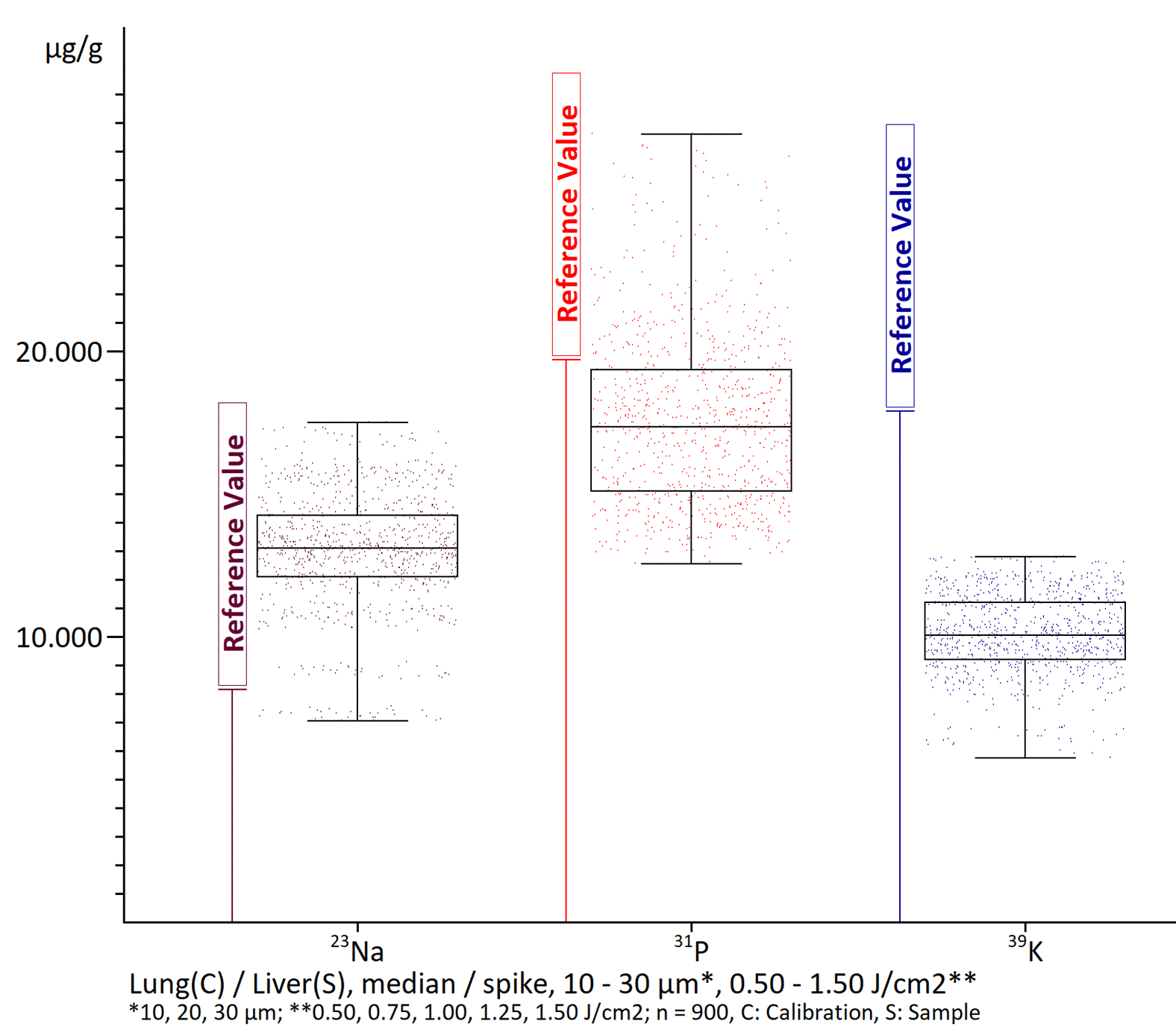

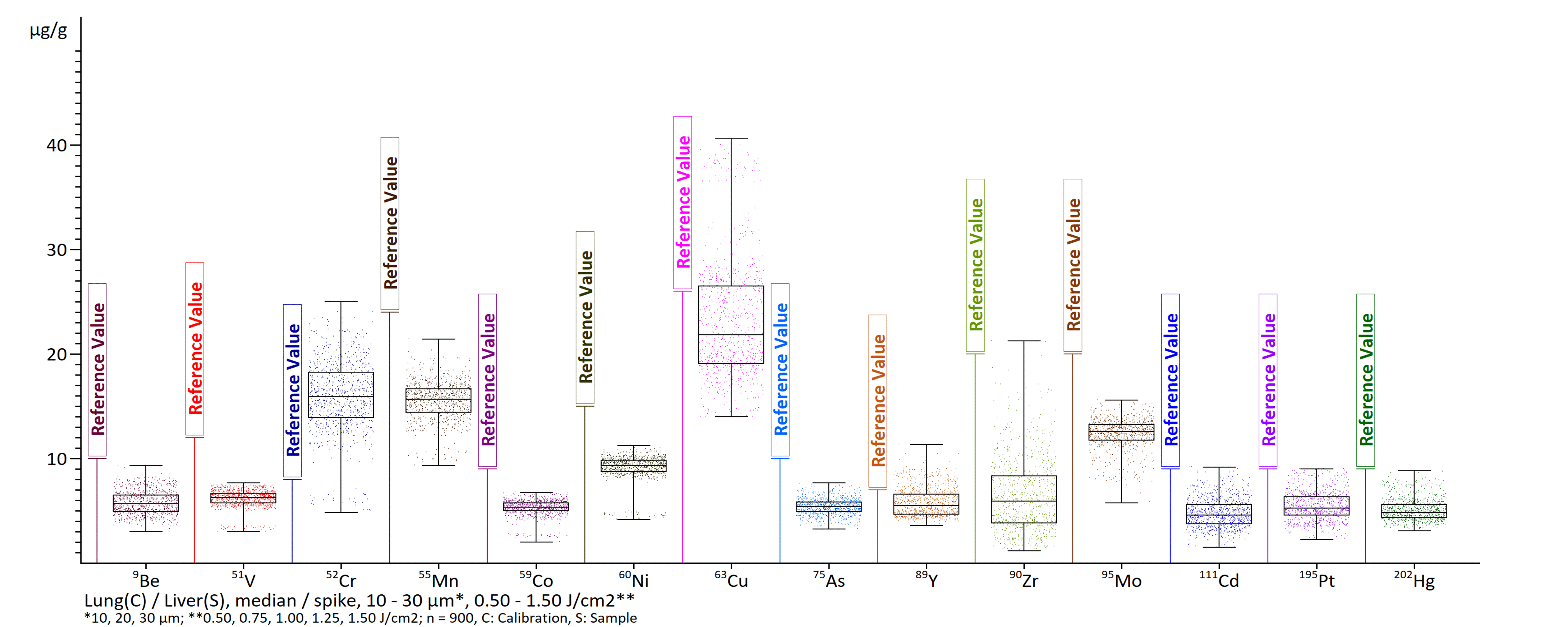


Evaluations (median/without de-spiking) of all measured isotopes in liver homogenate samples via liver homogenate calibrations

**Fig 3c.** The figure shows box-whisker plots of the median values of all measured isotopes in liver homogenate samples without de-spiking (element spikes) that were assessed using liver homogenate calibrations (n = 900). The top illustration represents isotopes within the lower concentration range (section 2.2.1). The lower left image shows isotopes in a range that is 10 times higher in concentration (27Al, 64Zn). The middle illustration below displays isotopes in a range that is 100 times higher in concentration (24Mg, 42Ca, 44Ca, 57Fe), while the lower right illustration shows isotopes in a range that is 1,000 times higher in concentration, including 23Na, 31P, and 39K.

C: Calibration, S: Sample


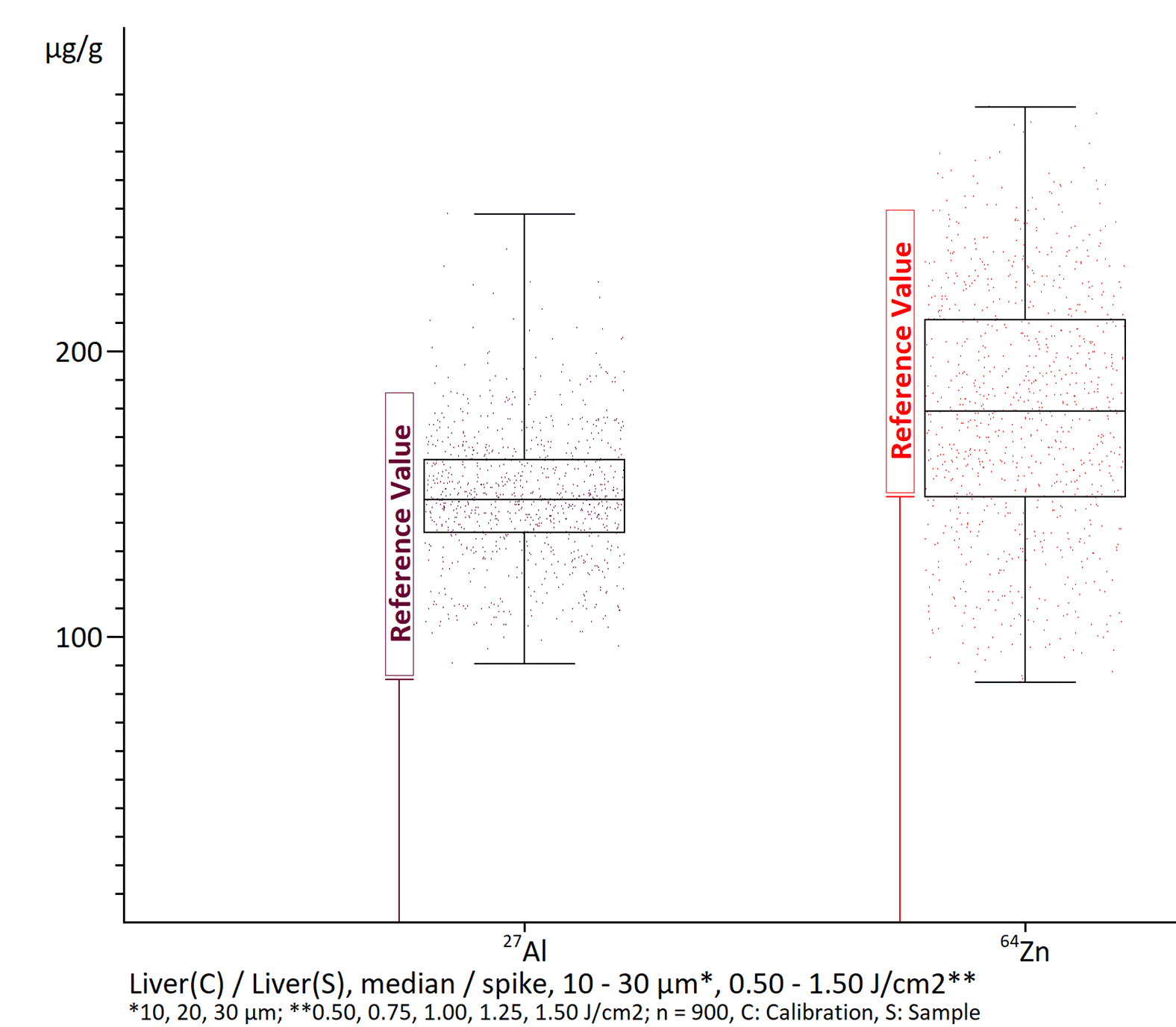

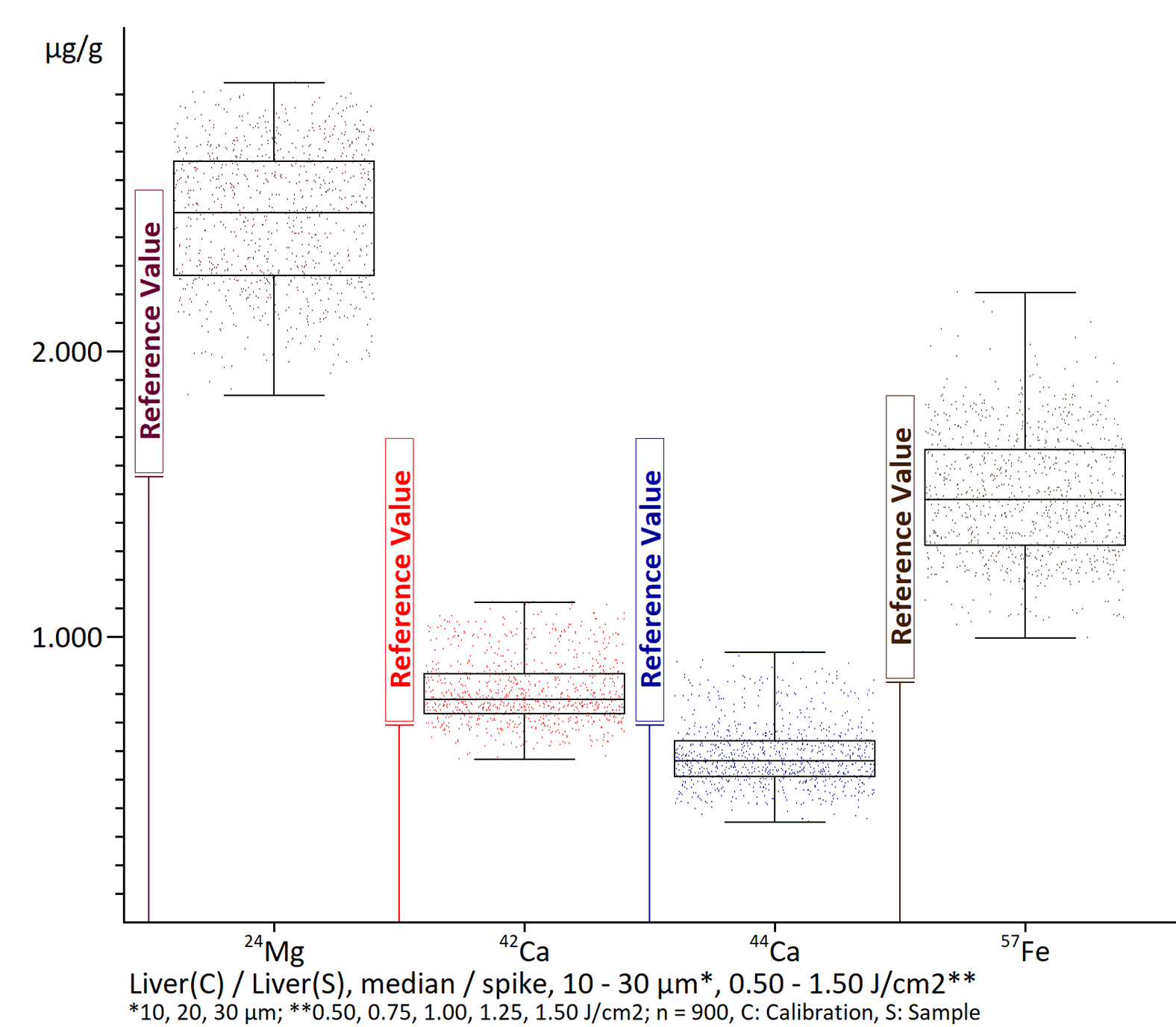

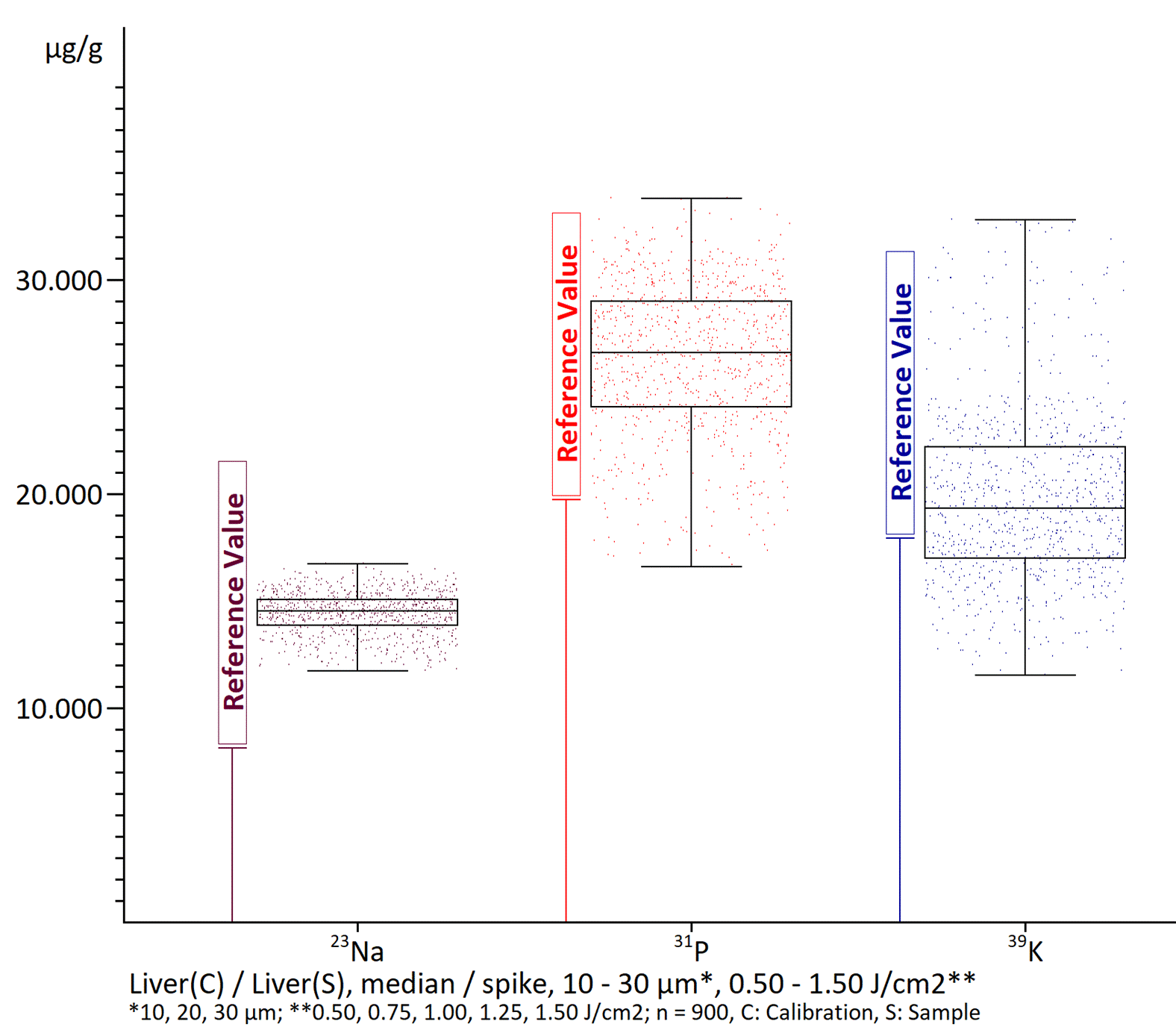

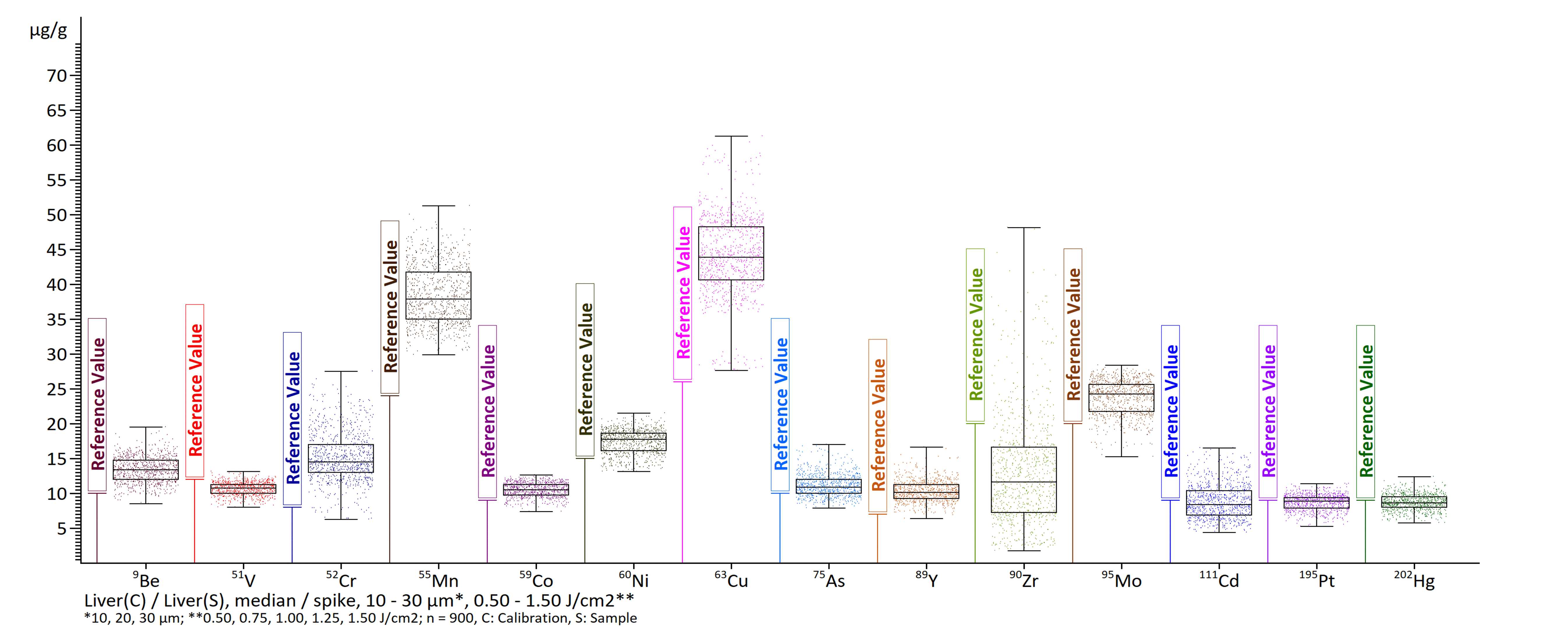

Supplement: Supplementary file 1 — Supplementary file1 (DOCX 38222 KB) [file 44211_2024_691_MOESM1_ESM.docx]
